# Constitutive alterations in vesicular trafficking increase the sensitivity of cells from celiac disease patients to gliadin

**DOI:** 10.1038/s42003-019-0443-1

**Published:** 2019-05-20

**Authors:** Giuliana Lania, Merlin Nanayakkara, Mariantonia Maglio, Renata Auricchio, Monia Porpora, Mariangela Conte, Maria Antonietta De Matteis, Riccardo Rizzo, Alberto Luini, Valentina Discepolo, Riccardo Troncone, Salvatore Auricchio, Maria Vittoria Barone

**Affiliations:** 1 Department of Translational Medical Science (Section of Pediatrics), University of Naples Federico II, Via S. Pansini 5, 80131 Naples, Italy; 2 European Laboratory for the Investigation of Food Induced Diseases (ELFID), University of Naples Federico II, Via S. Pansini 5, 80131 Naples, Italy; 3 Department of Molecular Medicine and Medical Biotechnology, University of Napoli Federico II, Via S. Pansini 5, 80131 Naples, Italy; 4 Telethon Institute of Genetics and Medicine, Via Campi Flegrei 34, 80078 Pozzuoli (NA), Italy; 5 Institute of Protein Biochemistry—IBP-CNR, Via Pietro Castellino 111, 80131 Naples, Italy

**Keywords:** Coeliac disease, Endosomes

## Abstract

Celiac Disease (CD) is an autoimmune disease characterized by inflammation of the intestinal mucosa due to an immune response to wheat gliadins. Some gliadin peptides (e.g., A-gliadin P57-68) induce an adaptive Th1 pro-inflammatory response. Other gliadin peptides (e.g., A-gliadin P31-43) induce a stress/innate immune response involving interleukin 15 (IL15) and interferon α (IFN-α). In the present study, we describe a stressed/inflamed celiac cellular phenotype in enterocytes and fibroblasts probably due to an alteration in the early-recycling endosomal system. Celiac cells are more sensitive to the gliadin peptide P31-43 and IL15 than controls. This phenotype is reproduced in control cells by inducing a delay in early vesicular trafficking. This constitutive lesion might mediate the stress/innate immune response to gliadin, which can be one of the triggers of the gliadin-specific T-cell response.

## Abstract (web summary)

Giuliana Lania, Merlin Nanayakkara et al. show that cells from celiac disease patients have delays in early endocytic trafficking and an increase sensitivity to gliadin peptide P31-43. Delaying early vesicular trafficking in control cells induces a celiac-like cellular phenotype, highlighting a role for the early-recycling endosomal system in celiac disease.

## Introduction

Celiac disease (CD) is an autoimmune disease characterized by an enteropathy with inflammatory and structural changes causing the remodeling of the small intestinal mucosa. These changes are due to the loss of oral tolerance to gluten, a protein contained in wheat, barley, and rye. The mucosal inflammation results from a Th1 response to gliadin peptides (e.g., the 33-mer A-gliadin peptide) presented by HLA-DQ2 or 8 (human leucocyte antigen)^1^ and activation of innate immune pathways. Several factors contribute to this activation, including other gliadin peptides, e.g., A-gliadin peptide P31-43^2^, not presented by HLA-DQ2 or 8^3^. Both the 33-mer and 25-mer (P31-55), containing the peptides P57-68 and P31-43, are not efficiently hydrolyzed by gastric, pancreatic, and intestinal proteases. Thus, these peptides are active in vivo in the intestine after gluten ingestion^4–6^. Major mediators of the proliferative and innate immune response of the celiac intestine to gliadin are IL(interleukin)-15^7,8^ and epidermal growth factor (EGF)^7,9,10^. P31-43 induces the innate immune response and enterocyte proliferation delaying the endocytic trafficking^7,11^. In both celiac enterocytes and CaCo-2 cells, P31-43 localizes to the early endosomes^10–12^. P31-43 shares sequence similarity to HGS (growth factor-regulated tyrosine kinase substrate) a key molecule involved in regulating endocytic maturation and located on the membranes of early endocytic vesicles^12^. P31-43, in CaCo-2 cells, interferes with the correct localization of HGS in early endosomes^12^. Therefore P31-43 induces two important effects, on one side it delays endocytic maturation and on the other side it alters the recycling pathway. A delay in endocytic maturation can reduce the degradation of epidermal growth factor receptor (EGFR), which is endocytosed in these vesicles. This delay prolongs its activation, with consequent increased proliferation, actin remodeling, and other biological effects. On the other side alterations in the recycling pathway direct more IL15R-α (IL15-receptor-α) to the cell surface, increasing trans presentation, in the epithelial cells of IL15/IL15R-α^7^. In CD, type 1 interferons also play a role in the loss of oral tolerance to gluten. In fact rotavirus infections are associated with an increased incidence of CD; IFN-α therapy can induce CD in some genetically susceptible individuals and IFN-α (interferon-α) expression is dysregulated in patients with CD^13^.

The reasons why P31-43 is dangerous to the CD small intestinal mucosa are unknown. Although effects on the endocytic pathway have been related to gliadin peptide P31-43, the reasons why this peptide is dangerous to the CD small intestinal mucosa are unknown.

Recently, constitutive activation of the EGFR-MAPK1 (mitogen-activated protein kinase 1)^14^ and NFκB (nuclear factor-κB)^15,16^ pathways and alterations in the cytoskeleton and adhesion^17,18^ have been observed in CD cells, together with differential expression of genes involved in cell adhesion, regulation of EGFR activation, small GTP (guanosine-5′-triphosphate) binding proteins, and autophagy^19–21^.

In the present study, we have shown that a constitutive alteration in vesicular trafficking in CD cells could render them more sensitive to the effects of gliadin peptides, this representing a predisposing condition to the damaging effects of gliadin peptides, such as P31-43.

## Results

### Endocytosis alteration in CD cells delayed EGFR decay

**Fig. 1:**
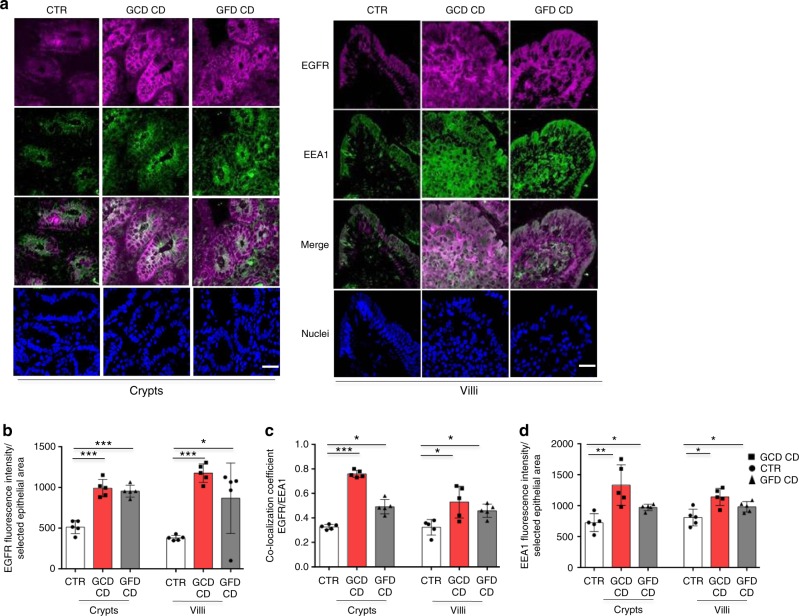
EGFR was localized more in the early vesicular compartment of enterocytes from patients with CD respect to controls. **a** Images of immunofluorescence staining for EGFR (magenta), EEA1 (green), and nuclei (blue) in crypts and villi of intestinal biopsies from patients with CD (GCD, gluten-containing diet; GFD, gluten-free diet) and controls (CTR). The white color in the merge panels indicates co-localization of EGFR and EEA1. Representative fields. Scale bar = 20 µm. **b** Statistical analysis of EGFR fluorescence intensity in selected epithelial areas. **c** Statistical analysis of the co-localization coefficients of EGFR with EEA1 staining. **d** Statistical analysis of fluorescence intensity of EEA1 staining in selected areas of crypt and villi enterocytes. One-way ANOVA Bonferroni corrected. *p < 0.05, **p < 0.01, ***p < 0.001

**Fig. 2:**
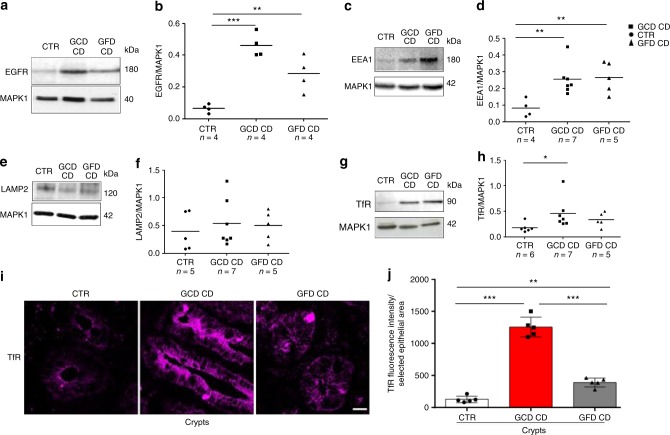
Levels of the EGFR, EEA1, LAMP, and TfR proteins in biopsies from patients with CD (GCD CD and GFD CD) and controls (CTR). Western blot (WB) of protein lysates and densitometry analysis of intestinal biopsies from several patients and controls, as indicated, blotted with anti-EGFR (**a**, **b**), anti-EEA1 (**c**, **d**), anti-LAMP2 (**e**, **f**), anti-TfR (**g**, **h**), and anti-MAPK1 antibodies used as a loading control. **i** Images of immunofluorescence staining for TfR in crypts in intestinal biopsies from patients with CD and controls. Representative fields. Scale bar = 20 µm. **j** Statistical analysis of fluorescence intensity of TfR-positive vesicles in crypts in selected epithelial areas. Samples from five patients and 5 controls were examined. Columns represent the means, and bars represent standard deviations; Student’s *t*-test, *p < 0.05, **p < 0.01, ***p < 0.001

EGFR, as measured by immunofluorescence (Fig. 1a, b) and Western blotting (Fig. 2a, b and Supplementary Fig. 1), was expressed at higher levels in CD enterocytes and biopsies (GCD CD *p* = 0.0001; GFD CD *p* = 0.0086) compared to control samples.

In celiac enterocytes, EGFR co-localized with the early vesicular marker EEA1 (early endocytic antigen) to a greater extent than controls (Fig. 1a, c) and with the late vesicular marker LAMP2 (lysosome-associated membrane protein 2) to a lesser extent than controls (Supplementary Fig. 2A, B).

**Fig. 3:**
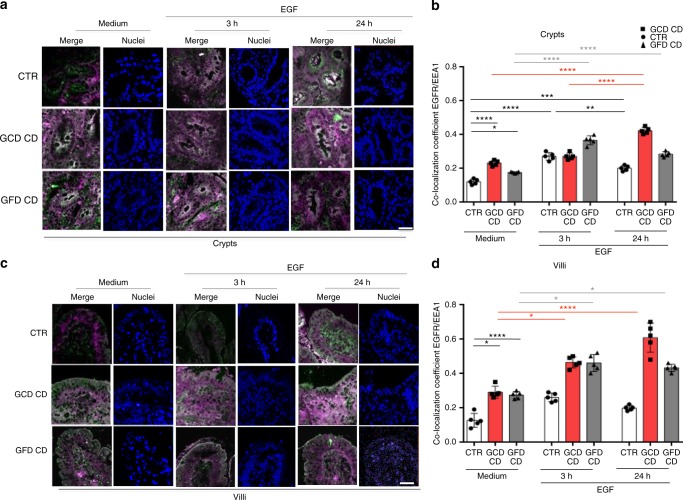
EGFR was localized more in the early vesicular compartment of enterocytes from patients with CD respect to controls after culture of small intestinal biopsies with EGF. Biopsies from patients with CD and controls were cultured in the presence of EGF to monitor the degradation of the EGF/EGFR complex. **a** Images of immunofluorescence staining of crypts from biopsies of patients and controls cultured in medium alone (Medium) for 24 h or in the presence of EGF for 3 or 24 h. The white color in the merge panels indicates co-localization of EGFR (magenta) and EEA1 (green). Representative fields. Scale bar = 20 µm. **b** Statistical analysis of the co-localization coefficient of EGFR with EEA1 in crypts as indicated. **c** Images of immunofluorescence staining of villi from biopsies of patients and controls cultured in medium alone (medium) for 24 h or in the presence of EGF for 3 or 24 h. The white color in the merge panels indicates co-localization of EGFR (magenta) and EEA1 (green). Representative fields. Scale bar = 20 µm. **d** Statistical analysis of the co-localization coefficient of EGFR with EEA1 in villi as indicated. In all experiments, five subjects in each group of patients, GCD CD, GFD CD and CTR, were examined. Three independent experiments were performed on samples from each group. Columns represent the means and bars represent standard deviations. One-way ANOVA Bonferroni corrected. *p < 0.05, **p < 0.01, ***p < 0.001

The decay of the EGFR was also studied in a dynamic way by cultivating the intestinal biopsies in the presence of the receptor ligand, the EGF. In these conditions we confirmed that in CD enterocytes the EGF/EGFR complex was delayed at the level of the early vesicular compartment (Fig. 3). In control crypt enterocytes, the co-localization of EGFR and EEA1 increased after a 3 h treatment compared to the medium sample and decreased after a 24 h treatment compared to the 3 h sample (Fig. 3a, b). In contrast, in CD crypts enterocytes, EGFR co-localization with EEA1 increased after a 3 h EGF treatment and remained elevated at 24 h (Fig. 3a, b). Similar results were obtained with villi (Fig. 3c, d).

Very low amounts of EGF labeled with the fluorescent marker Alexa Fluor-488 are present in the control biopsies at both 3 and 24 h after chase, indicating that the complex was degraded. However, Alexa Fluor-488-labeled EGF was still present in the crypt enterocytes from patients with CD on a GCD or GFD, after 3 and 24 h of chase (Supplementary Fig. 3A, B).

Moreover, both the fluorescence intensity and levels of the EEA1 (Figs. 1d and 2c, d), but not LAMP2 (Fig. 2e, f and Supplementary Fig. 1) protein were increased in CD enterocytes and in CD biopsies (GCD CD *p* = 0.001; GFD CD *p* = 0.0095) from patients with CD on a GCD or GFD compared to controls. Levels of TfR (transferrin receptor) were increased in the proliferating crypt compartment of CD biopsies (*p* = 0.047), as expected^22^ (Fig. 2g–j and Supplemental Fig. 1).

Based on these results, an alteration in the endocytic compartment is present in CD enterocytes, delaying the decay of EGFR at the level of the early endocytic compartment.

**Fig. 4:**
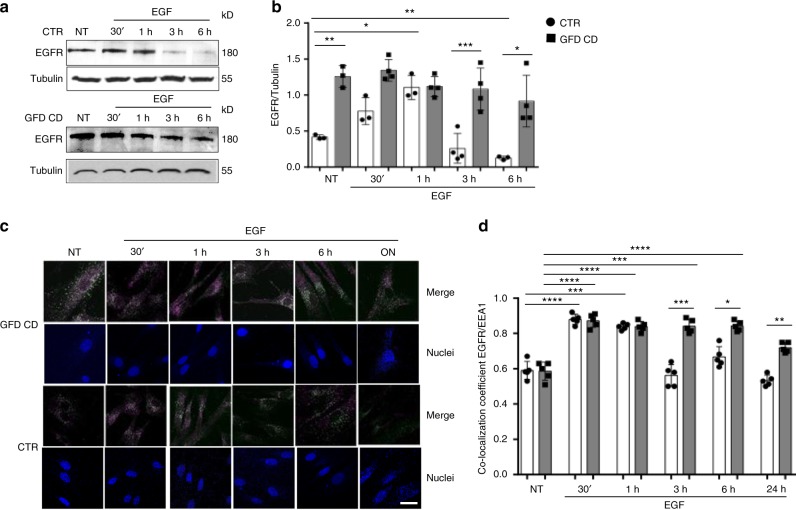
In CD skin fibroblasts EGFR was delayed in the early vesicular compartment. Levels of the EGFR protein and co-localization of EGFR/EEA1 in CD fibroblasts compared to control fibroblasts both before treatment (NT) and after EGF treatment at the indicated times. **a** WBs of protein lysates from NT fibroblasts and cells treated with EGF for 30′, 1, 3, or 6 h blotted with anti-EGFR and anti-tubulin antibodies, as loading control. Representative blots. **b** Densitometry analysis. One-way ANOVA Bonferroni corrected. *p < 0.05, **p < 0.01, ***p < 0.001. **c** Images of immunofluorescence staining. The white color in the merge panels indicates co-localization of EGFR (magenta) and EEA1 (green) and nuclei are blue. Representative fields. Scale bar = 20 µm. **d** Statistical analysis of the co-localization coefficient of EGFR with EEA1 staining. Greater amount of EGFR co-localized with EEA1-positive vesicles in CD fibroblasts compared to controls at 3, 6, and 24 h after EGF treatment. In all experiments, samples from 5 patients and 5 controls were tested. Columns represent the means, and bars represent standard deviations. One-way ANOVA Bonferroni corrected. *p *<* 0.05, **p < 0.01, ***p < 0.001

To confirm the constitutive alteration of the endocytic pathway in CD cells we studied EGFR trafficking in skin fibroblasts, in a very different environment from the intestine, the main inflammatory site in CD, using immunofluorescence staining and Western blotting. Fibroblasts were treated with EGF for different times up to 24 h (Fig. 4). Higher levels of the EGFR protein were present in GFD CD fibroblasts compared to controls before and after EGF treatment (Fig. 4a, b and Supplementary Fig. 4). Experiments assessing the co-localization of EGFR with EEA1 (Fig. 4c, d), LAMP2 (Supplementary Fig. 5A) and TfR (Supplementary Fig. 6A) revealed a delay in the trafficking of EGFR in the early endosomes in GFD CD fibroblasts compared to controls. Co-localization with the recycling marker TfR was increased in GFD CD fibroblasts compared to controls, but only at later time points (Supplementary Fig. 6A).

**Fig. 5:**
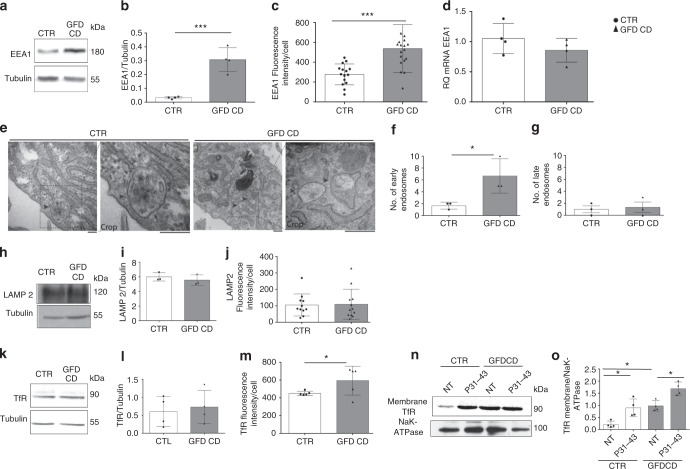
Early, late, and recycling vesicular compartments in CD and CTR skin fibroblasts. **a**, **b** Levels of the EEA1 protein in CD and CTR fibroblasts. Representative WB (**a**) and densitometry analysis (**b**) of protein lysates from fibroblasts obtained from patients with CD and controls blotted with anti-EEA1 and anti-tubulin, as a loading control. **c** Statistical analysis of the fluorescence intensity of EEA1 staining/cell. **d** Quantitative PCR analysis of the EEA1 mRNA in CD fibroblasts compared to controls. Columns represent the means and bars represent the standard deviations of a representative experiment performed in triplicate. Similar results were obtained from 4 patients and 4 controls. **e**–**g** Electron microscopy analysis of fibroblasts from controls and CD patients. Representative images and statistical analysis of fibroblasts from 3 patients and 3 controls. Arrows indicate early endosomes in controls and CD biopsies. Scale bars = 100 nm. **h**, **i** Levels of the LAMP2 protein in CD and CTR fibroblasts. Representative WB (**h**) and densitometry analysis (**i**) of protein lysates from fibroblasts obtained from patients with CD and controls blotted with anti-LAMP2 antibodies, and anti-tubulin antibodies, as a loading control. **j** Statistical analysis of fluorescence intensity of LAMP2 staining/cell. **k**, **l** Levels of the TfR protein in CD and CTR fibroblasts. Representative WB (**k**) and densitometry analysis (**l**) of protein lysates from fibroblasts obtained from patients with CD and controls blotted with anti-TfR antibodies, and anti-tubulin antibodies, as a loading control. **m** Statistical analysis of fluorescence intensity of TfR staining/cell. Samples from five patients and 5 controls were analyzed in all experiments, unless stated otherwise. Columns represent the means, and bars represent the standard deviations. Student’s *t*-test, *p < 0.05, ***p < 0.001. **n**, **o** TfR levels in the membrane fraction of CD fibroblasts compared with CTR samples with and without P31-43 treatment for 3 h. **n** WB analysis of proteins from membrane fractions of CTR and CD fibroblasts blotted with anti-TfR and NaK-ATPase antibodies. Representative blots. **o** Densitometry analysis of WB of samples from 4 patients and 4 controls. Columns represent the means, and bars represent the standard deviations. One-way ANOVA Bonferroni corrected. *p < 0.05

Levels of the EEA1 protein (*p* = 0.0007) (Fig. 5a–c and Supplementary Fig. 7), but not mRNA (Fig. 5d), were increased in CD fibroblasts, indicating that the increased level of the EEA1 protein was due to delayed degradation rather than increased synthesis. An electron microscopy analysis (Fig. 5e–g) confirmed the increase in the number of early, but not late, vesicles in CD fibroblasts. In contrast, similar levels of the LAMP2 (Fig. 5h–j and Supplementary Fig. 7) and TfR proteins (Fig. 5k–o and Supplementary Fig. 7) were observed in CD fibroblasts and controls. Interestingly, TfR levels in the membrane fractions were increased in CD fibroblasts compared to controls, suggesting that the recycling pathway is also altered in these cells (Fig. 5n, o and Supplementary Fig. 7). In fact, pulse-chase experiments with Alexa Fluor-488-labeled Tf showed an increase in Tf uptake in CD fibroblasts compared to controls (Supplementary Fig. 8).

In CD fibroblasts, the trafficking of EGF-Alexa Fluor-488 was also delayed (Supplementary Fig. 9A, B), but the uptake of EGF-Alexa Fluor-488 was not different from the controls (Supplementary Fig. 9C).

In summary, an alteration in the endocytic compartment was observed in fibroblasts from patients with CD on GFD, with a delay in the trafficking from the early to the late vesicles.

**Fig. 6:**
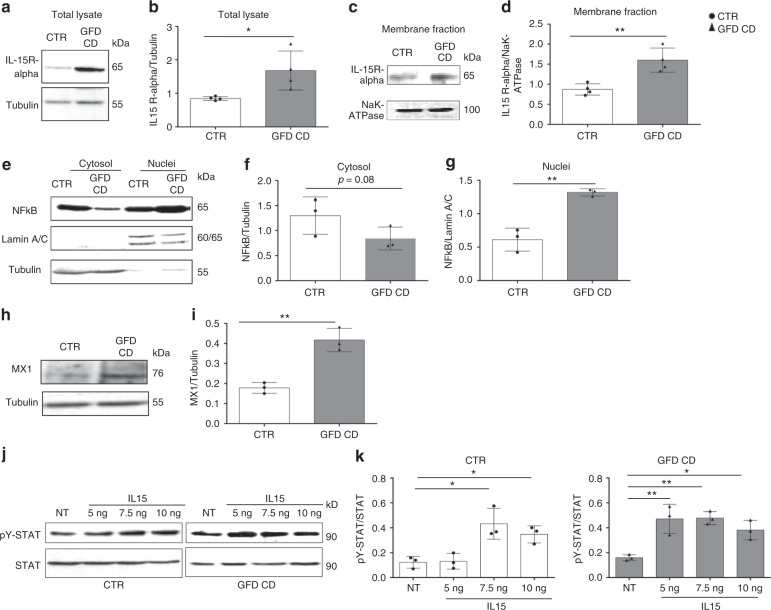
Levels of the innate immunity markers IL15R-α and MX1 and the inflammation marker nuclear NFκB are increased in CD skin fibroblasts. **a**, **b** Levels of IL15R-α in the total lysates. WB analysis of proteins from total lysates of CTR and CD fibroblasts blotted with anti-IL15R-α and anti-tubulin antibodies, as indicated. Representative blots (**a**) and densitometry analysis (**b**) of WBs of samples from 4 patients and 4 controls. **c**, **d** Levels of IL15R-α in the membrane fractions. WB analysis of proteins from membrane fractions of CTR and CD fibroblasts blotted with anti-IL15R-α and anti-NaK-ATPase antibodies, as indicated. Representative blots (**c**) and densitometry analysis (**d**) of WBs of samples from 4 patients and 4 controls. **e**–**g** WB analysis of protein lysates from the nuclei and cytosol of CTR and CD fibroblasts blotted with anti-NFκB, anti-lamin A/C (nuclei loading control), and anti-tubulin (cytosol loading control) antibodies, as indicated. Representative blots (**e**) and densitometry analysis (**f**, **g**) of WBs of samples from 3 patients and 3 controls. **h** WB analysis of proteins from total lysates from CTR and CD fibroblasts blotted with anti-MX1 and anti-tubulin antibodies, as indicated. Representative blots. **i** Densitometry analysis of WBs of samples from 3 patients and 3 controls. Student’s *t*-test compared to the control (CTR) sample, *p < 0.05, **p < 0.01, ***p < 0.001. **j**, **k** Dose response curve of STAT 5 phosphorylation in response to treatment with increasing IL15 concentrations (5, 7.5, or 10 ng/ml) for 30 min. **j** WB analysis of proteins from total lysates of CTR and CD fibroblasts blotted with anti-STAT 5 and anti-pY STAT 5 antibodies, as indicated. Representative blots. **k** Densitometry analysis of WBs of samples from 3 patients and 3 controls. Columns represent the means and bars represent standard deviations. One-way ANOVA Bonferroni corrected. *p < 0.05, **p < 0.01

Cellular markers of stress/innate immunity are constitutively increased in CD cells and biopsies^7,8,14,23–28^. We found also in fibroblasts, that not only the EGFR (Fig. 2a, b) but also the IL15R-α levels were increased (*p* = 0.029). IL15R-α levels were increased particularly in the membrane fractions (*p* = 0.0046) where it can be transpresented (Fig. 6a–d and Supplementary Fig. 10). NFκB and IFN-α pathways were also activated, as evidenced by increased levels of nuclear NFκB (*p* = 0.0024) (Fig. 6e–g and Supplementary Fig. 10) and MX1 (*p* = 0.003) (myxovirus resistance gene A) (Fig. 6h, i and Supplementary Fig. 10). These findings confirmed that the fibroblasts isolated from skin explants were a good model for studying the celiac cellular phenotype. Interestingly in fibroblasts derived from CD duodenal biopsies from GCD and GFD we found, respect to controls, the same alterations of the skin fibroblasts: EGFR colocalized more with EEA1 and less with LAMP2; EGFR and EEA1 levels were increased as well as innate immunity (MX1 and STAT5) and inflammation markers (NFκB) (Supplementary Figs. 11, 12)^29,30^.

We evaluated the activation of the downstream IL15R-α effector, STAT 5 (signal transducer and activator of transcription 5) by treating CD fibroblasts with increasing IL15 concentrations to investigate the effects of the increased IL15R-α levels on the functions of these cells. Low concentrations of IL15 (5 ng) were sufficient to induce STAT 5 phosphorylation in CD fibroblasts, but not in control cells (Fig. 6j, k and Supplementary Fig. 10), indicating that CD cells are more sensitive to IL15, a major mediator of the innate immune response in patients with CD.

We hypothesized that CD fibroblast expressed constitutively activated NFκB and MX1, because of a delay of the early endocytic trafficking. To determine the correlation between alterations of the endocytic trafficking to the activation of EGFR, IL15R-α, IFN-α, and NFκB in celiac fibroblasts we induced, in control fibroblasts, a delay in early endocytic trafficking by silencing HGS, a key regulator of early to late endocytic trafficking^31^. Circa 70% of the HGS protein was silenced (Supplementary Figs. 13, 14). In these conditions it is known that HGS silencing could increase EGFR/EEA1 colocalization and EGFR levels^31^. Moreover HGS silencing induced in control fibroblasts an increase of the IL15R-α and MX1 protein levels and NFκB phosphorylation (Supplementary Figs. 13, 14), reproducing the constitutive alterations found in CD fibroblasts. Finally HGS silencing increased levels of the EEA1 protein (Supplementary Figs. 13, 14).

### Celiac and siHGS cells were more sensitive to gliadin peptide

We have then studied the effect of gliadin peptide P31-43 treatment on intracellular vesicular trafficking of biopsies and fibroblasts from CD patients and controls, to verify, on one side, whether in control cells the celiac alterations of the endocytic trafficking was reproduced by P31-43 treatment and on the other side if the celiac cells were more sensitive than controls to the effect of gliadin.

**Fig. 7:**
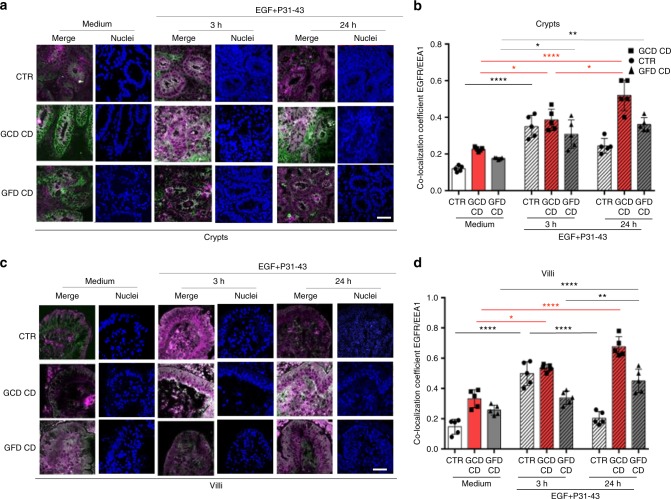
Treatment with P31-43 induced in enterocytes a transient delay of the EGF/EGFR trafficking in controls and a prolonged delay in CD patients. EGF/EGFR trafficking after P31-43 in controls and CD biopsies. **a** Images of immunofluorescence staining of crypts in biopsies from patients and controls that had been cultured in medium alone for 24 h (medium) or in the presence of EGF and P31-43 for 3 or 24 h. The white color in the merge panels indicates co-localization of EGFR (magenta) and EEA1 (green). Nuclei are in blue. Representative fields. Scale bar = 20 µm. **b** Statistical analysis of the co-localization coefficient of EGFR with EEA1. **c** Images of immunofluorescence staining of villi in biopsies from patients and controls that had been cultured in medium alone for 24 h (medium) or in the presence of EGF and P31-43 for 3 or 24 h. The white color in the merge panels indicates co-localization of EGFR (magenta) and EEA1 (green). Nuclei are in blue. Representative fields. Scale bar = 20 µm. **d** Statistical analysis of the co-localization coefficient of EGFR with EEA1. In all experiments, five subjects from each group of patients (GCD CD and GFD CD) and controls (CTR) were examined. Three independent experiments were performed on samples from each group. Columns represent the means, and bars represent standard deviations; hatched columns indicate P31-43 treatment. One-way ANOVA Bonferroni corrected. *p < 0.05, **p < 0.01, ***p < 0.001

In cultured control biopsies, treatment with the A-gliadin peptide P31-43 reproduced the celiac alterations delaying EGFR trafficking in the early/recycling endocytic compartment of crypt and villi enterocytes. Co-localization of EGFR with EEA1 after EGF treatment was actually increased by 3 h treatment with P31-43, but decreased after a 24 h treatment suggesting a transitory delay of the vesicular trafficking (Fig. 7). In CD enterocytes, the co-localization of EGFR with EEA1 increased after a 3 h treatment and remained elevated at 24 h (Fig. 7). Showing that in the presence of P31-43 CD enterocytes presented a persistent reduced ability to dispose of the EGF load in the early endocytic vesicles.

**Fig. 8:**
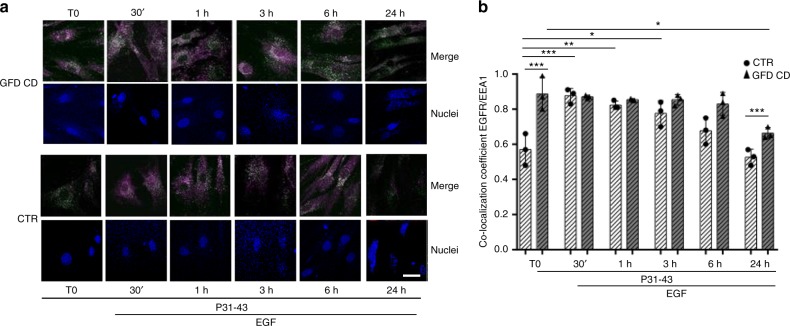
Treatment with P31-43 induced in skin fibroblasts a transient delay of the EGF/EGFR trafficking in controls and a prolonged delay in CD patients. EGF/EGFR trafficking after P31-43 in control and CD fibroblasts. **a** Images of immunofluorescence staining for EGFR and EEA1 in skin fibroblasts from patients and controls treated with P31-43 for 10′ (T0) and then with both EGF and P31-43 for 30 min, 1, 3, 6, or 24 h. The white color in the merge panels indicates co-localization of EGFR (magenta) and EEA1 (green), nuclei are in blue. Representative fields. Scale bar = 20 µm. **b** Statistical analysis of the co-localization coefficient of EGFR with EEA1. In all experiments, samples from five subjects from each group of patients (GCD CD and GFD CD) and controls (CTR) were examined. Three independent experiments were performed using samples from each group. Columns represent the means, and bars represent standard deviations; hatched columns indicate P31-43 treatment. One-way ANOVA Bonferroni corrected. *p < 0.05, **p < 0.01, ***p < 0.001

Treatment with P31-43 induced a delay in EGFR trafficking in EEA1-positive vesicles also in control and CD fibroblasts (Fig. 8 and Supplementary Fig. 5C). Interestingly, in GFD-CD fibroblasts treated with P31-43 alone (T0, Fig. 8), EGFR/EEA1 co-localization was increased compared to the untreated sample (NT, Fig. 4d, *p* < 0.05), unlike the CTR fibroblasts. In control fibroblasts, treatments with EGF and P31-43 induced a significant increase in EGFR/EEA1 co-localization (at 30′, 1 and 3 h) that decreased to the T0 levels after 24 h suggesting a transient delay of the vesicular trafficking. In contrast, in EGF and P31-43-treated fibroblasts from patients with CD on a GFD, the increased EGFR/EEA1 co-localization persisted for up to 24 h, and at this time the difference was statistically significant compared to the control fibroblasts.

The analysis of the recycling compartment revealed that TfR levels were increased in the membrane fraction both in control and CD fibroblasts after P31-43 treatment, indicating that the peptide also interfered with the recycling compartment (Fig. 5n, o). In P31-43-treated control fibroblasts, the increase in EGFR co-localization with the recycling marker TfR was transient (Supplementary Fig. 6B). In GFD CD, co-localization increased at all time points compared to control fibroblasts (Supplementary Fig. 6B). Thus, in the presence of P31-43, the celiac fibroblasts tried to clear the EGF/EGFR cargo by redirecting it to the recycling compartment.

Treatment of control fibroblasts with P31-43 also increased the levels of the EEA1, EGFR, and IL15R-α proteins and activated the NFκB and IFN-α pathways, reproducing the constitutive alterations observed in untreated CD cells (Supplementary Figs. 13, 14).

**Fig. 9:**
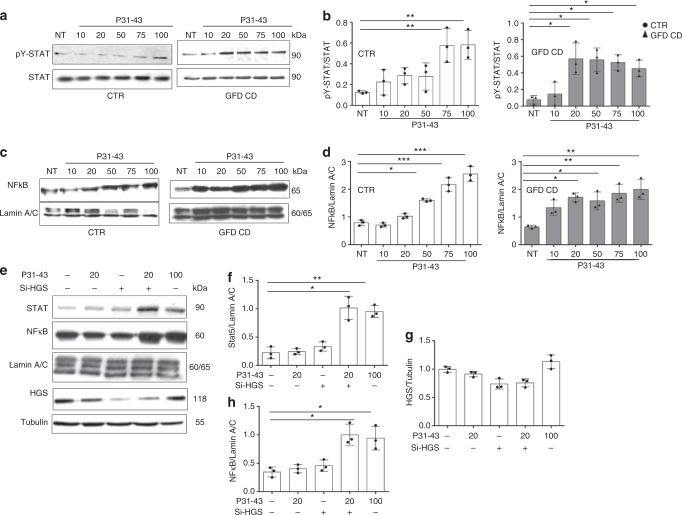
Celiac fibroblasts and siHGS control fibroblasts were more sensitive to the P31-43 treatment than control cells. **a**, **b** Dose response curve of STAT 5 phosphorylation in response to treatment with increasing P31-43 concentrations (10, 20, 50, 75, or 100 µg/ml) for 30 min. **a** WB analysis of proteins from total lysates from CTR and CD fibroblasts blotted with anti-STAT 5 and anti-pY STAT 5 antibodies, as indicated. Representative blots. **b** Densitometry analysis of WBs of samples from 3 patients and 3 controls. Columns represent the means and bars represent standard deviations. **c**, **d** Dose response curve of nuclear NFκB in response to treatment with increasing P31-43 concentrations (10, 20, 50, 75, or 100 µg/ml) for 30 min. **c** WB analysis of proteins from nuclear fraction, from CTR and CD fibroblasts blotted with antibodies anti-NFκB and anti-lamin A/C, used as nuclear proteins loading control, as indicated. Representative blots. **d** Densitometry analysis of WBs of samples from 3 patients and 3 controls. Columns represent the means, and bars represent standard deviations. **e**–**h** Cooperation of low doses of siHGS and P31-43 on STAT 5 and NFκB activation in control cells. STAT 5 and NFκB nuclear fraction in response to treatment with inactive (20 µg/ml) or active (100 µg/ml) doses of P31-43 in CTR fibroblasts before and after silencing HGS (25 ng siHGS). **e** WB analysis of proteins from cytosol fraction blotted with antibodies anti-HGS, anti-tubulin (citosol loading control), and of proteins from nuclear fraction blotted with antibodies anti-STAT 5 and anti-NFκB and anti-lamin A/C (nuclear loading control), as indicated. Representative blots are shown. **f**–**h** Densitometry analysis of WBs of samples from 3 controls. Columns represent the means, and bars represent standard deviations. One-way ANOVA Bonferroni corrected. *p < 0.05, **p < 0.01, ***p < 0.001

CD fibroblasts were more sensitive to low concentrations of P31-43 than controls, as determined using both STAT 5 and NFκB activation as read outs of the innate immunity and the inflammatory response, respectively. As shown in Fig. 9a–d (Supplementary Fig. 15), low amounts (20 µg/ml) of P31-43 induced STAT 5 and NFκB activation in CD fibroblasts, but not in controls. To reproduce the gluten sensitivity of CD fibroblasts in CTR cells we have used low doses (25 ng/ml) of silencing HGS (25% decrease of the protein) that by them self were not effective to induce activation of STAT 5 and NFκB. In these conditions the CTR fibroblasts became more sensitive to low concentrations of P31-43, in fact 20 µg/ml of P31-43, normally ineffective on CTR cells, induced STAT 5 and NFκB activation. (Fig. 9e–h). Very low doses of silencing HGS (12 ng/ml) were able to further activate Stat5 and NFκB in GFD-CD (see Supplementary Figs. 16, 17) and to induce sensitivity to very low doses of P31-43 (10 µg/ml) normally ineffective in CD-GFD fibroblasts. Based on these data, the celiac cells were more sensitive to the effect of the gliadin peptide P31-43 than controls, as evidenced by (a) their increased sensitivity to low concentrations of the peptide and (b) the prolonged delay in EGF/EGFR trafficking in the early endocytic compartment and redirection to the recycling compartment in CD cells. The sensitivity of CD cells to low concentrations of P31-43 was reproduced in HGS-silenced control cells.

## Discussion

In patients with CD, signals provided by the distressed epithelium have been assumed to be required for intraepithelial cytotoxic lymphocyte-mediated tissue destruction^32^, with exposure to gluten necessary to maintain the disease. Unresolved inflammation, organelle dysfunction and other cellular and metabolic stresses underlie the development of chronic inflammatory diseases associated with aging, including obesity, diabetes, cardiovascular diseases, asthma, and cancer^33–36^.

In the present study, we found in CD fibroblasts, from skin and duodenal biopsies, an increase of markers of the innate immune response (EGFR, IL15R-α, MX1) and of the inflammatory response (NFkB). CD cells (enterocytes and fibroblasts) presented also a constitutive alteration in the intracellular vesicular system at the level of the early-recycling compartment. In fact, in CD enterocytes and fibroblasts, the numbers of early vesicles were increased, and the EGF/EGFR trafficking was delayed in the early endocytic vesicles; moreover, the decay of the EGFR was prolonged, EEA1 and TfR levels were increased.

CD fibroblasts were more sensitive to low concentrations of IL15 and the gliadin peptide P31-43, and P31-43 caused in CD fibroblasts a more prolonged delay in EGF/EGFR trafficking in the early endocytic and recycling compartments compared to the control. CD cells were therefore not only inflamed but also more prone to inflammation induced by gliadin. This finding could explain why P31-43, which delays early to late vesicle trafficking, damages CD cells. Interestingly P31-43 treatment reproduced only transiently the celiac cellular phenotype in control enterocyte and fibroblast.

At this point we hypothesized that most of the alterations described in the CD cells were due to a delay of the endocytic trafficking. To demonstrate this we induced in control fibroblasts a delay of the early endocytic trafficking by transfecting siHGS. HGS is a key regulator of endocytic maturation, its silencing induces delayed decay of EGFR and delay of the maturation of early to late vesicles^7,12^. Notably, in our control fibroblasts siHGS was able per se to reproduce the constitutive alterations found in the celiac cells, such as the increased levels of the EGFR and EEA1 proteins and the markers of innate immunity (IL15R-α and MX1) and the inflammatory (NFκB) response. Confirming that the morphological and functional alterations in the intracellular vesicular system observed in CD cells were linked to the inflammatory and immune/mediated pathogenesis of the gluten-dependent disease

Moreover to further demonstrate that the increased sensitivity of the CD cell to the P31-43 was due to the delay of the vesicular trafficking we have delayed the endocytic trafficking in CTR cells by transfecting low doses of silencing HGS, that by themselves were not effective to induce activation of STAT 5 and NFκB. In these conditions, siHGS rendered the control fibroblasts sensitive to low concentrations of P31-43 activating the innate immunity and inflammation markers.

Recently, several human diseases characterized by inflammation and/or autoimmunity have been attributed to alterations in vesicular trafficking at various levels^37,38^. In particular, hereditary autoimmune/mediated lung disease in humans was found to be due to alterations in vesicular trafficking, revealing a role for intracellular transport in the induction of human autoimmunity^39^.

Finally, we analyzed whether non-HLA loci and/or published eQTL (expression quantitative trait loci) effects associated with CD were involved in vesicular trafficking. Interestingly, 3 of 42 non-HLA loci and 5 of 127 cis eQTL effects that have previously been shown to be related to the immune response or other functions were also related to the vesicular trafficking (Table 1). In particular, 3 of these genes (ATXN2, ataxin2; TRAPPC4, trafficking protein particle complex 4; and MP1, melanisation protease 1) were involved in regulating EGFR/MAPK1 trafficking and activation, 3 (ATXN2, TRAPPC4 and ERP29, endoplasmic reticulum protein 29) were involved in endocytosis, and 4 (GLB1, galactosidase, beta 1; FYCO1, FYVE and coiled-coil domain-containing 1; TRAPPC4 and ULK, Unc-51 like autophagy activating kinase 1) were involved in the autophagosome/lysosome trafficking (Table 1)^40–50^. The results of the analysis of the non-HLA loci and/or published eQTL effects support a role for vesicular trafficking in the pathogenesis of CD.

**Table 1:** Non-HLA loci and eQTLs effects associated with CD tagged for vesicular trafficking

	Reported candidate genes^30^	Array studies^30^	RNA-Seq studies^30^	Tissue of origin^30^	Function/localization in endo-membrane system
**AP4B1**	**X**		**X**	Blood	The gene encodes for the beta subunit of the adaptor complex 4 (AP-4) complex. The AP-4 complex is not associated with clathrin and is involved in TGN-to-endosome trafficking and polarized sorting^41^.
**GLB1**	**X**			Blood	The gene encodes for beta galactosidase, a lysosomal enzyme that hydrolyzes terminal beta-linked galactose residue from ganglioside substrates and other glycoconjugates. Its downregulation is associated to the lysosomal function and to cellular autolysis^42,43^.
**ATXN2**	**X**			Biopsies (GCD GFD CD)	• This gene encodes ataxin2, a cytoplasmic protein localized to the endoplasmic reticulum and plasma membrane, and involved in endocytosis, and mTOR signaling. • It has been found in complex with EGFR, can regulate EGFR density on the cell surface and delays its degradation^44^.
**FYCO1**		**X**		Blood	• This gene encodes for a protein (containing a RUN domain, FYVE-type zinc finger domain and GOLD domain) that is involved in autophagy and interacts with PI3P and the small GTPase Rab7. • Participates to the transport of the late endosomes and autophagosomes mediated by microtubules. • Transport adaptor^45^
**TRAPPC4**		**X**		Blood	• The gene encodes for a component of the multimolecular complex Transport Particle TRAPP, which controls several trafficking steps along the exocytic and autophagic pathway. • Interacts with pYERK and transports it to the nuclei^46^.
**ERP29**		**X**		Thymus	• This gene encodes for an ER protein belonging to the family of PDIs, which facilitates the folding of selected newly synthesized protein and is involved in their trafficking at the early secretory pathway. It is involved in the epithelial–mesenchymal transition. • Its over-expression induces epithelial differenziation (mesenchymal/epithelial transition). On the contrary its downregulation induces epithelial dedifferentiation with loss of tight junctions^47^.
**MP1**		**X**	**X**		• This gene encodes for a mannose phosphate isomerase. • N-Glycosylation as determinant of epidermal growth factor receptor conformation in the membranes^48^. • MPI inhibition reduces FGFR family signaling^49^.
**ULK3**		**X**	**X**	Blood	The gene encodes for the Unc1 like kinase 3, a kinase involved in autophagy^21^ and in cytokinesis through the phosphorylation of the ESCRTIII complex^50^.

In conclusion, CD is an intestinal inflammatory autoimmune disease caused by a combination of a multigene profile and environmental factors, including dietary gluten. In the present study, we described a celiac cellular phenotype characterized by a constitutive alteration in intracellular vesicular trafficking that rendered the cells sensitive to the inflammatory response to gliadin peptides.

## Methods

### Ethical statements

All adult subjects provided written informed consent for the use of their biopsies in this study. Parents or tutors provided written informed consent for subjects aged less than 18 years. The protocol for this study was approved by the Ethical Committee of the University “Federico II”, Naples, Italy (ethical approval, C.E. n. 230/05).

### Organ culture studies

For organ culture studies, biopsy fragments from the duodenum were obtained from patients with CD presenting with villous atrophy (12 patients, mean age of 5 years), controls affected by gastroesophageal reflux (12 subjects, mean age of 6 years) and patients with CD on a GFD (10 subjects, mean age of 12 years). The age range for all patients was 2–16 years. Patients with CD on a GFD had negative serology (anti-tTg antibody titers ranging between 0 and 1.5 U/ml and negative for antiendomisum antibodies (EMA)) and a normal biopsy (Marsh T0). Patients with villous atrophy (Marsh T3c) had positive serology (anti-tTg antibodies >50 U/ml and EMA positive). Anti-tTg antibody titers were measured using Eurospital kit EU-tTG (Supplementary Table 1). Patients had been on a GFD for at least 4 years, the adherence to GFD was complete and patients had remission of the symptoms. None of the CD patients was affected by dermatitis herpetiformis. Biopsy fragments were cultivated as previously described^10^. Briefly intestinal samples were cultured for 24 h with medium alone or with 100 µg/ml P31-43 (INBIOS, Naples, Italy), 100 ng/ml EGF (Invitrogen, San Giuliano Milanese, Italy) and EGF-Alexa Fluor-488 (20 ng/ml, Invitrogen, San Giuliano Milanese, Italy). Specimens were harvested, snap-frozen in liquid nitrogen, embedded in OCT and stored at −80 °C until required for further analyses.

### Immunofluorescence staining of biopsies

We used double immunofluorescence staining to evaluate EGFR and EEA1 levels and co-localization. The 5-µm cryostat sections from biopsies were air dried, fixed with acetone (10 min), and stained with rabbit anti-EGFR (1005 sc-03, Santa Cruz Biotechnology, CA, USA) and anti-EEA1 (C-15 sc-6414 Santa Cruz Biotechnology, CA, USA) antibodies for 1 h at room temperature, after blocking with 1% bovine serum albumin (BSA) in PBS for 15 min at room temperature (RT). Then, the sections were washed (3 times, 5 min each) with PBS at RT, and fluorescein-conjugated anti-rabbit (for EGFR) (1:50; SC-03, Santa Cruz Biotechnology, CA, USA) and anti-goat secondary antibodies (for EEA1) (1:50; Santa Cruz Biotechnology, CA, USA) were applied for 45 min at room temperature in a dark box to avoid bleaching. After washes with PBS, sections were counterstained with Hoechst (Sigma-Aldrich, Milan, Italy), mounted with Mowiol (Sigma-Aldrich, Milan, Italy) and observed under a Zeiss LSM510 or Zeiss LSM700 confocal microscopes (laser scanning microscope) (Germany). The co-localization analysis was performed using previously reported methods^12^ (Supplementary Methods). Images were obtained with a 63× objective unless differently stated.

### Primary fibroblast culture

Fibroblasts were cultured from skin biopsies obtained from patients with CD (GFD) and controls. We obtained fibroblasts from seven patients with CD on a gluten-free diet (ages ranged from 17–43 years) and seven healthy controls (ages ranged from 25–30 years). Patients had been on a gluten-free diet for at least 4 years and showed normal biopsies (Marsh T0), serum anti-tTg antibody titers ranging between 0 and 1.5 U/ml and EMA negativity. Fibroblasts were cultured also from duodenal biopsies obtained from patients with CD (GFD and GCD) and controls. We obtained fibroblasts from 5 CD patients on a GFD (ages ranged from 4–11 years), 5 GCD patients (ages ranged from 4–11 years) and healthy no CD controls (ages ranged from 4–11 years). Patients had been on a gluten-free diet for at least 4 years and showed normal biopsies (Marsh T0), serum anti-tTg antibody titers ranging between 0 and 1.5 U/ml and EMA negativity. The adherence to GFD was complete, all patients had remission of the symptoms. GCD patients showed altered biopsies (Marsh T3c), serum anti-tTg antibodies >50 U/ml and EMA positive. None of the CD patients was affected by dermatitis herpetiformis. The fibroblasts were tested for mycoplasma contamination (MycoFluor™ Mycoplasma Detection Kit M7006, Invitrogen, Milan, Italy). For details see Supplemental Material.

### Fibroblasts immunofluorescence staining

Samples were prepared and analyzed as described in Supplementary Methods.

### Western blot

Biopsy fragments (5 mg wet weight each) from duodenum obtained from GCD and GFD CD patients and controls were homogenized in 100 µl tissue homogenization buffer (50 mM Tris–HCl [pH 8], 150 mM NaCl, 5 mM MgCl_2_, 1% Triton, 0.5% sodium deoxycholate, 0.1% SDS, 1 mM PMSF, 1 mM VO4, Aprotinin, and LAP; all purchased from Sigma, Milan, Italy, except for LAP, which was purchased from Roche, Milan, Italy) using a 2-ml conical Wheaton glass tube with a Teflon pestle. For details of WB procedures see Supplemental Methods.

### HGS silencing

The fibroblasts were cultivated in medium alone or in the presence of P31-43 as required. We used the HiPerfect kit (Qiagen) according to the manufacturer’s instructions to transfect si HGS (50 ng, 25 ng, 12 ng siHGS). Silencing experiments were performed with two different silencing mRNAs for HGS (Hs HGS 5 and Hs HGS 6) (Qiagen GmbH, D-40724 Hilden, GE). The two silencing gave similar results, only Hs HGS 5 is shown in the figures.

### mRNA analysis

Total RNA was extracted from fibroblasts from patients with CD on a GFD and controls using TRIZOL reagent (Ambion®-Life Technologies). The mRNA concentration was measured using a Nanodrop® spectrophotometer, and subsequently, the RNA quality was analyzed using agarose gel electrophoresis in Tris/Borate/EDTA buffer (TBE, Sigma, Milan, Italy), as described previously^18^. The RNA (1 µg) was reverse transcribed into cDNAs using the High Capacity cDNA Reverse Transcription kit, according to the manufacturer’s protocol. Experiments were performed with approximately 40 ng of cDNA templates, according to the manufacturer’s protocol (TaqMan® Gene Expression Assay), using a 7900HT Fast Real-Time PCR system. The gene expression assay used for EEA1 gene is Hs00929215_m1 (Life Technologies), and the probe is located on Chr 12 exons 28–29 (caaga ttggatctac agaaaaaatc tgaagccctt gaaagtatca agcaaaagct taccaagcaa gaggaagaaa aacaaatcct gaaacaagat tttgaaactt taagtcaaga aacaaagatt). The expression of each gene was normalized to the expression of an endogenous housekeeping gene (B2M). Relative quantification was performed using the ΔΔCt method. SDS software (ABI, version 1.4 or 2.4) was used to analyze the raw data, and subsequently, an additional statistical analysis was performed using GraphPad Prism 5.01® software.

### EM analysis

Samples were prepared and analyzed as described in Supplementary Methods^51^.

### Statistical analysis

GraphPad Prism 5.lnk software (GraphPad Software, San Diego, CA, USA) was used for statistical analyses and to construct graphical representation. The statistical analysis of the differences was performed using Student’s *t*-test. A *p* value < 0.05 was considered statistically significant. Two-tailed comparison was used for all the statistical analysis. The sample size was chosen after considering the variance of the control samples and the number of samples needed to assess the extent of the expected effect was estimated to be 3 or 4. Therefore, the chosen samples size was 3, 4 and more often 5. Multiple comparison was done with ANOVA Bonferroni corrected analysis as stated in the figures.

### Reporting summary

Further information on research design is available in the Nature Research Reporting Summary linked to this article.

## Supplementary information

Supplementary Information

Supplementary Data 1

Reporting Summary

## Data Availability

All data generated or analyzed during this study are included in this published article (and its Supplemental Information files). Data that have generated the statistics are available in the Supplementary Data 1 file. All other relevant data are available from the authors.
