# Author Correction: Constitutive alterations in vesicular trafficking increase the sensitivity of cells from celiac disease patients to gliadin

**DOI:** 10.1038/s42003-020-0906-4

**Published:** 2020-04-02

**Authors:** Giuliana Lania, Merlin Nanayakkara, Mariantonia Maglio, Renata Auricchio, Monia Porpora, Mariangela Conte, Maria Antonietta De Matteis, Riccardo Rizzo, Alberto Luini, Valentina Discepolo, Riccardo Troncone, Salvatore Auricchio, Maria Vittoria Barone

**Affiliations:** 1 Department of Translational Medical Science (Section of Pediatrics), University of Naples Federico II, Via S. Pansini 5, 80131 Naples, Italy; 2 European Laboratory for the Investigation of Food Induced Diseases (ELFID), University of Naples Federico II, Via S. Pansini 5, 80131 Naples, Italy; 3 Department of Molecular Medicine and Medical Biotechnology, University of Napoli Federico II, Via S. Pansini 5, 80131 Naples, Italy; 4 Telethon Institute of Genetics and Medicine, Via Campi Flegrei 34, 80078 Pozzuoli (NA), Italy; 5 Institute of Protein Biochemistry—IBP-CNR, Via Pietro Castellino 111, 80131 Naples, Italy

**Keywords:** Coeliac disease, Endosomes

Correction to: *Communications Biology* 10.1038/s42003-019-0443-1, published online 20 May 2019.

In the original published version of this article the grant number attributed to the Italian Society for Celiac Disease (Associazione Italiana Celiachia; AIC) was incorrectly given as Project No. 053_FC_2013. The correct grant number is Grant 005_FC_2016, awarded to Monia Porpora. The error has been corrected in the HTML and PDF versions of the article.

