# Supplementary material for: Constitutive alterations in vesicular trafficking increase the sensitivity of cells from celiac disease patients to gliadin
Source: Commun Biol. 2019 May 20;2:190. doi: 10.1038/s42003-019-0443-1 (PMC6527696; doi:10.1038/s42003-019-0443-1)
Supplement: Supplementary file 1 — Supplementary Information [file 42003_2019_443_MOESM1_ESM.pdf]

Supplemental Material

Supplementary figures

Supplementary figure I

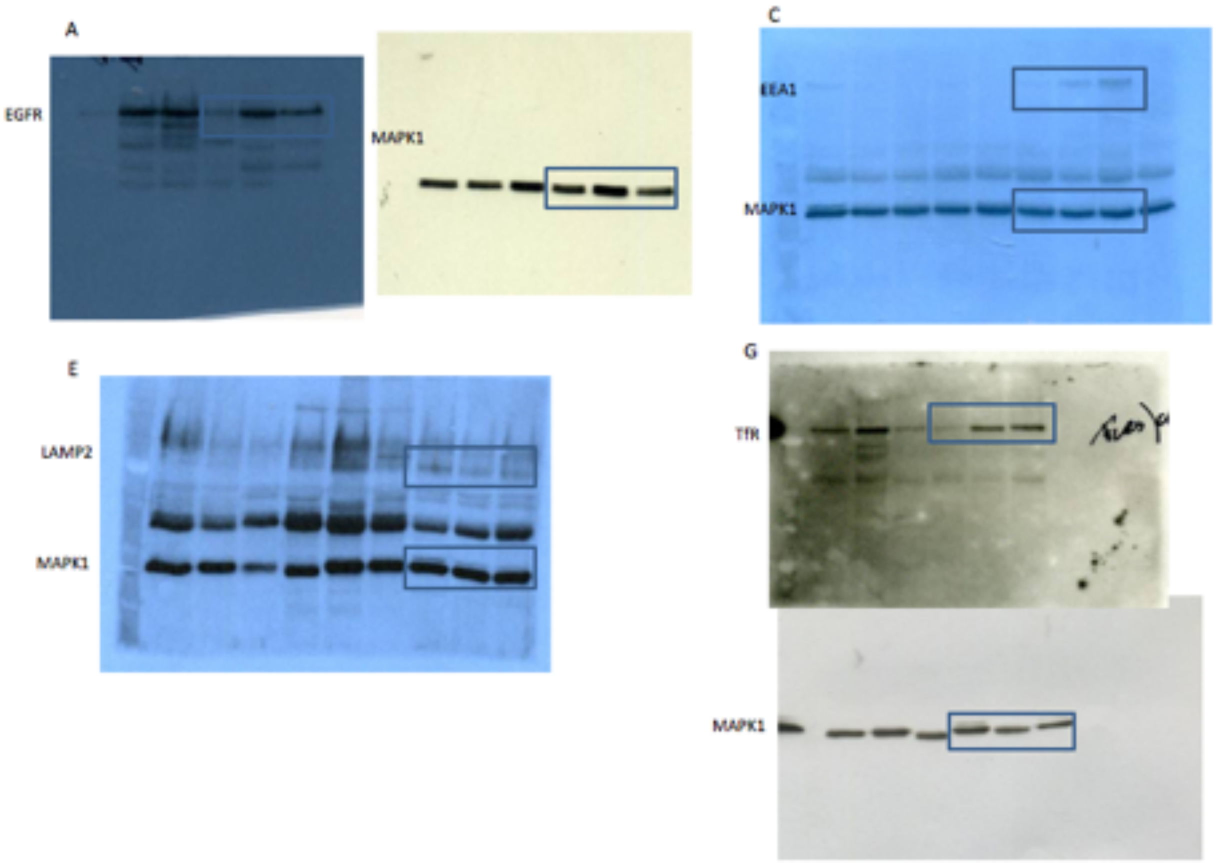

Supplementary figure I: Un-cropped Blots from figure 2. The letters (A, C, E, G) on the un-cropped gels refers to those of the figure 2.

Supplementary figure 2

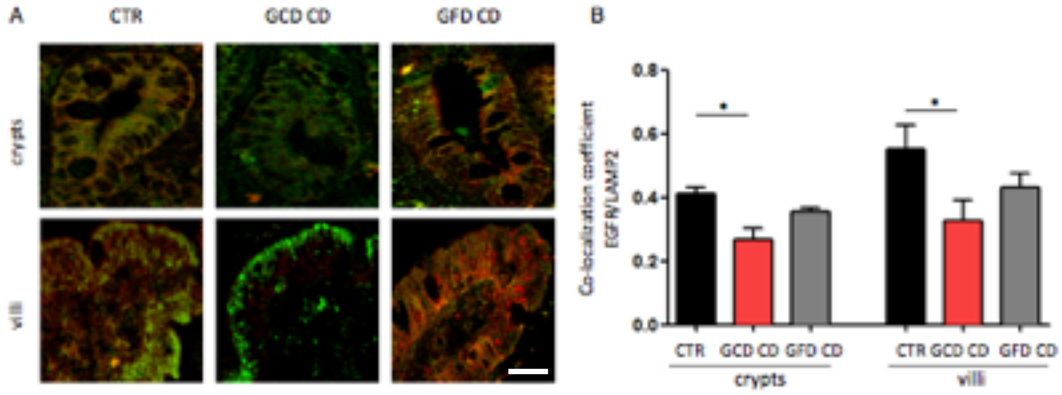

Supplementary figure 2

Localization of EGFR in the late vesicular compartments in enterocytes from patients with CD and control biopsies. Co-localization of EGFR with the late endocytic marker LAMP2. Less EGFR co-localized with LAMP2-positive vesicles in crypts and villi enterocytes from patients with CD than in controls. A. The yellow colour in the merge panels indicates co-localization of immunofluorescence staining for EGFR (red) and LAMP2 (green) in crypts and villi of biopsies from patients and controls. Representative fields are shown. Scale bar = 20µm. B) Less EGFR co-localized with LAMP2-positive vesicles in crypts and villi enterocytes from patients with CD than in controls. Statistical analysis of the co-localization coefficient of EGFR with LAMP2 staining. In all experiments, samples from five subjects in each group of patients (GCD CD and GFD CD) and controls (CTR) were examined. Three independent experiments were performed using samples from each group. Columns represent the means, and bars represent standard deviations. Student's t-test: * $P<0.05$.

Supplementary figure 3

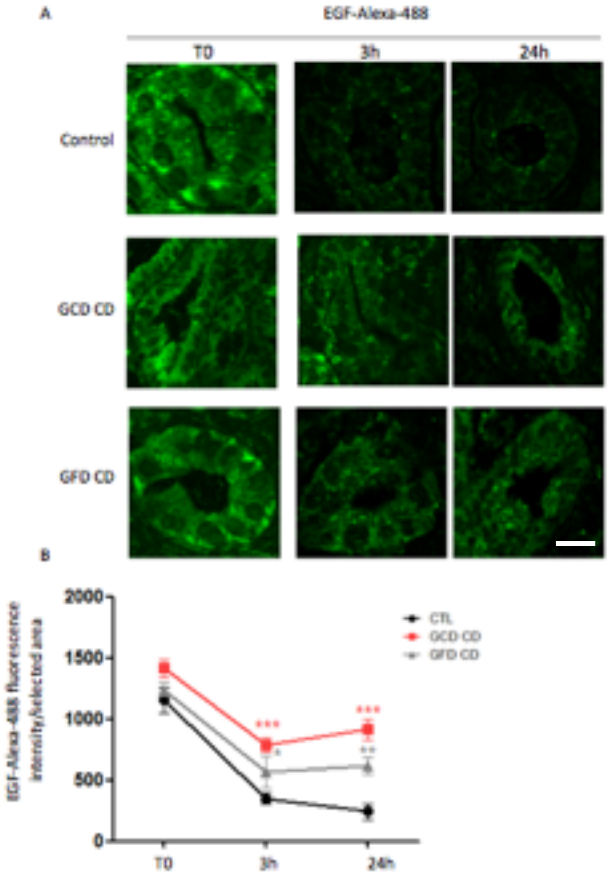

Supplementary figure 3

Biopsies from patients with CD and controls cultured in the presence of EGF-Alexa Fluor-488.

EGF-Alexa Fluor-488 trafficking was delayed in the endocytic compartment of CD enterocytes in both the acute and the remission phases of the disease. A. Images of immunofluorescence maker EGF-Alexa-488 in crypts. Biopsies from patients with CD on a GCD or GFD and controls (CTR) were cultured with EGF-Alexa Fluor-488 for 3 h (T0) pulse and chased with unlabelled EGF for 3

or 24 h to monitor the degradation of the EGF/EGFR complex. Images of crypts were obtained with a 63x objective (2x digital zoom). The results from one representative experiment are shown. Scale bar = 20μm. B. Statistical analysis of fluorescence intensity of EGF-Alexa Fluor-488 in crypts from intestinal biopsies. Greater amounts of EGF-Alexa Fluor-488 were still present in CD enterocytes after 3 and 24 h of chase than in control cells. In all experiments, samples from five subjects in each group of patients (GCD CD and GFD CD) and controls (CTR) were examined. Three independents experiments were performed using samples from each group. Bars represent standard deviations. Student's t-test compared to the control: * $P<0.05$, ** $P<0.01$, *** $P<0.001$.

Supplementary figure 4

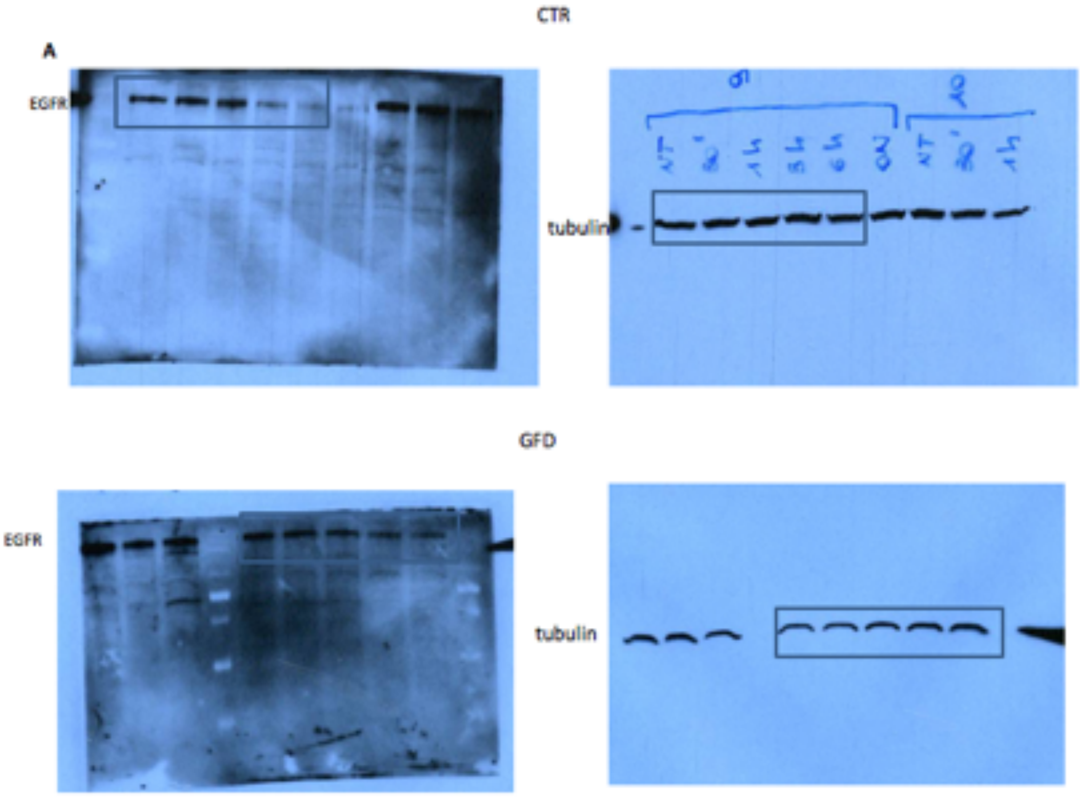

Supplementary figure 4: Un-cropped Blots from figure 4. The letter (A) on the un-cropped gels refers to that of the figure 4.

Supplementary figure 5:

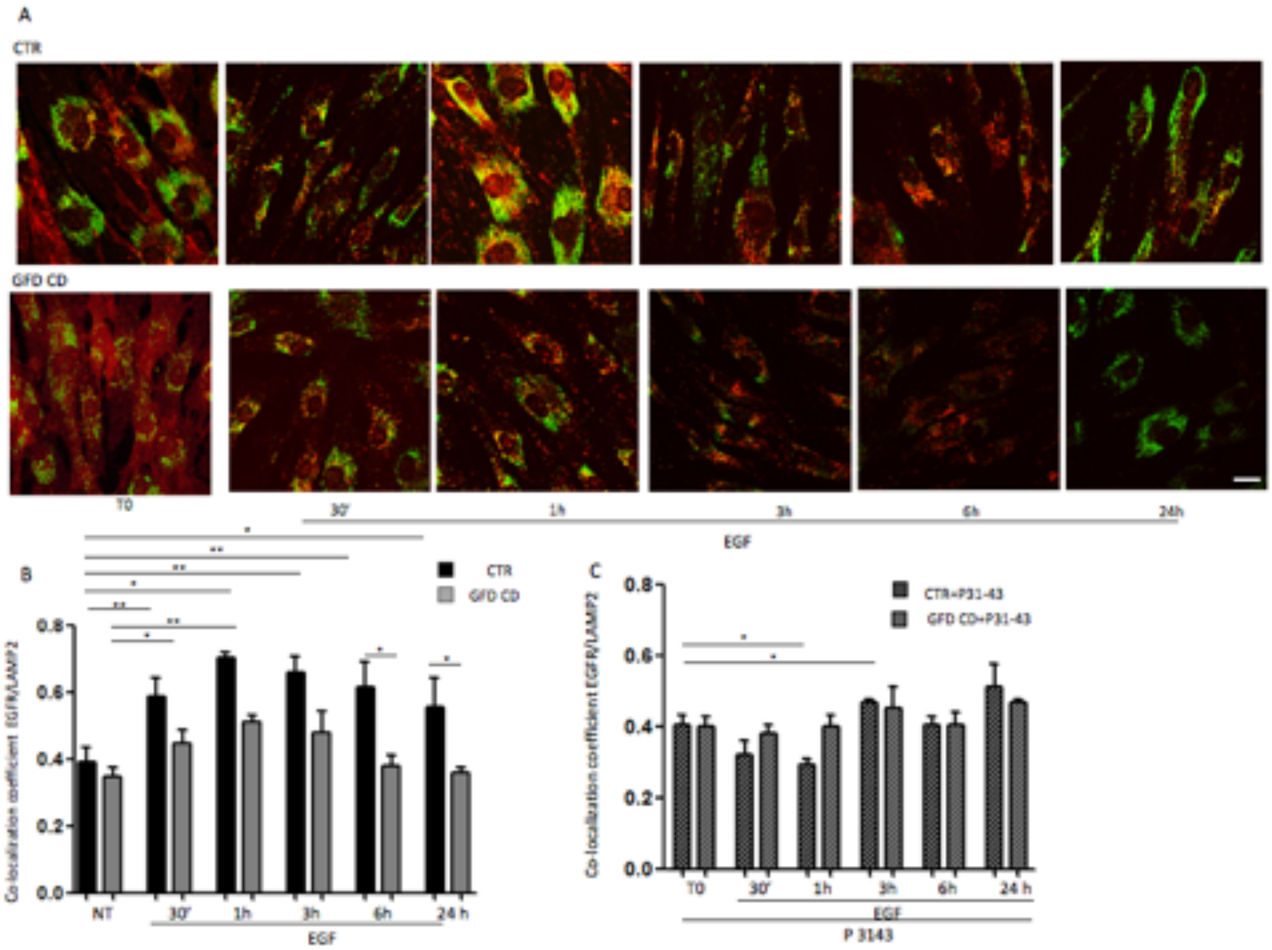

Supplementary figure 5:

EGF induced EGFR endocytic trafficking in late vesicular compartments in skin fibroblasts from patients with CD and controls cultured in absence or presence of P31-43.

A. Less EGFR co-localized with LAMP2-positive vesicles in CD fibroblasts than in control fibroblasts at all time points after EGF treatment. The yellow colour in the merge panels indicates co-localization of EGFR (red) with late endocytic marker LAMP2 (green) in fibroblasts from patients and controls treated with EGF at the indicated times. Representative fields are shown. Scale bar = 20µm. B. In both control and CD fibroblasts, co-localization of EGFR with the late endocytic marker LAMP2 increased at 30 min and 1 h compared to the not treated sample (NT) and decreased at later time points. EGFR/LAMP co-localization was decreased at all time points in CD fibroblasts compared to CTR fibroblasts. These results suggested a delay in EGFR trafficking in the early endocytic compartment in CD fibroblasts. C. EGF induced EGFR trafficking in the late endocytic compartment in fibroblasts from patients with CD and controls after treatment with P31-43 for 10’ (T0) and then with both EGF and P31-43 for 30 min, 1 h, 3 h, 6 h or 24 h. An increase in EGFR co-localization with LAMP2 was not observed in control fibroblasts after 30 min or 1 h of P31-43 treatment compared to the untreated control fibroblasts (B), as expected from a delay in EGFR trafficking in the early endocytic compartment. No variation in EGFR-LAMP2 co-localization was observed in CD fibroblasts treated with P31-43.

Supplementary figure 6:

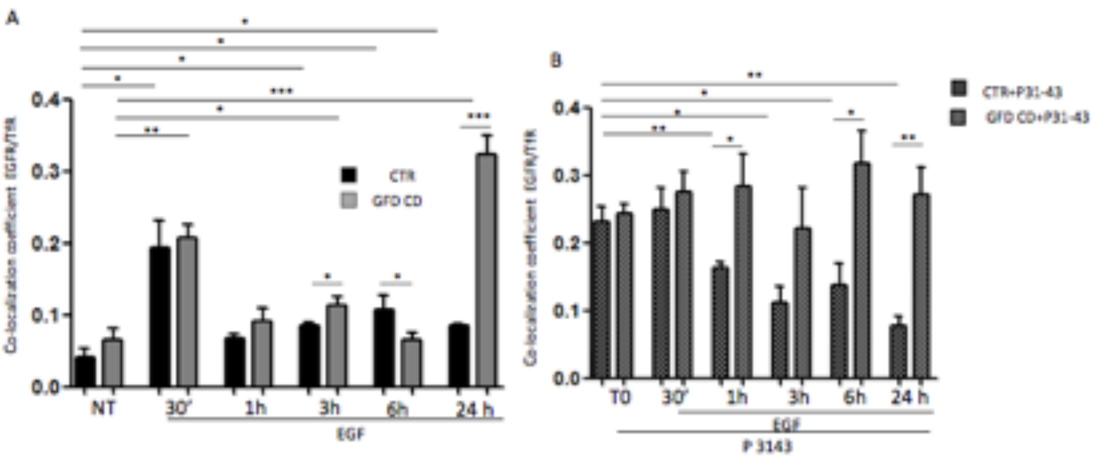

Supplementary figure 6: **Co-localization of EGFR with the recycling endocytic marker TfR in fibroblasts treated with and without P31-43**. A. A greater amount of EGFR co-localized with TfR in CD fibroblasts compared to control cells after a 24 h treatment with EGF. Statistical analysis of the co-localization coefficient of EGFR with TfR staining. EGF treatment induced EGFR/TfR co-localization in fibroblasts from patients with CD and controls at 30 min. At 24 h, a significant increase in EGF levels was observed in recycling compartments in cells from patients with CD compared to controls. B. EGF induced EGFR trafficking in recycling compartments in fibroblasts from patients with CD and controls after treatment with P31-43 for 10’ (T0) and then with both EGF and P31-43 for 30 min, 1 h, 3 h, 6 h or 24 h. In control fibroblasts, a marked decrease in EGFR/TfR co-localization was observed beginning at 30 min that was similar to the samples treated with EGF alone. In contrast, in CD fibroblasts, a greater amount of EGFR co-localized with TfR at all time points after EGF treatment in presence of P31-43. In all experiments, samples from 5 subjects in each group of patients and controls were examined. Three independents experiments were performed using samples from each group. Columns represent the means and bars represent standard deviations. Student’s t-test: * $P<0.05$, ** $P<0.01$, *** $P<0.001$.

Supplementary figure 7

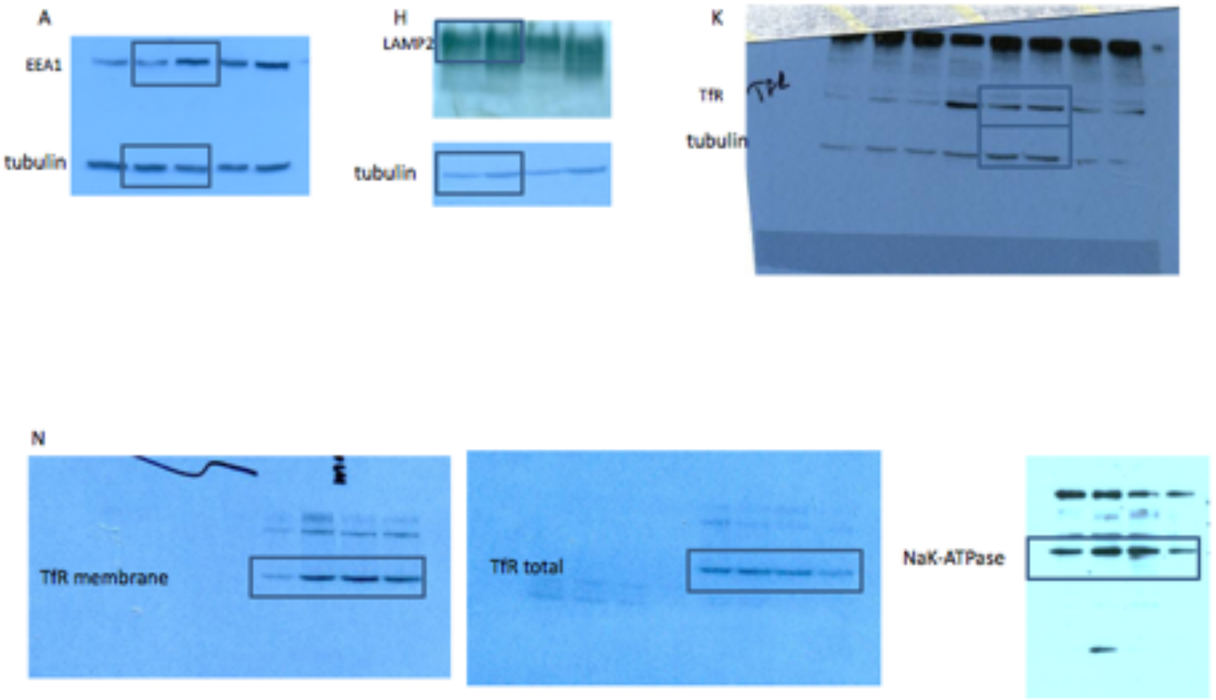

Supplementary figure 7: Un-cropped Blots from figure 5. The letters (A, H, K,N) on the un-cropped gels refers to that of the figure 5.

Supplementary figure 8:

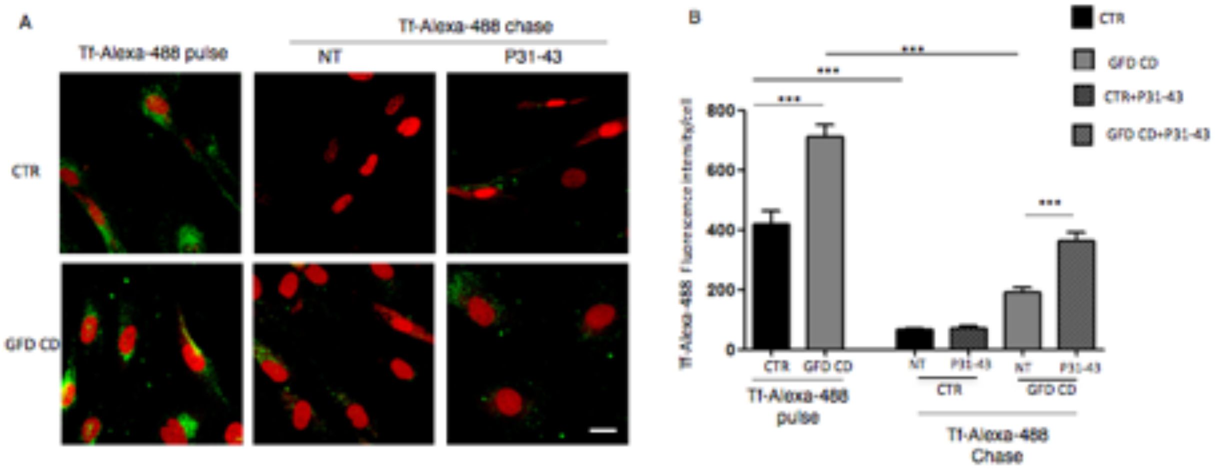

Supplementary figure 8:

Transferrin recycling in fibroblasts from patients with CD on a GFD and controls. The uptake of transferrin-Alexa Fluor-488 (Tf-Alexa Fluor-488) was increased in CD fibroblasts compared to control cells, and in the presence of P31-43, its recycling was delayed. Fibroblasts were treated with Tf-Alexa Fluor-488 (Tf pulse) for 1 h with or without P31-43, and then after washing, cells were cultivated in medium alone for 1 h (Tf chase) to promote Tf recycling. A. Images of immunofluorescence staining for Tf-Alexa Fluor-488 (green) in fibroblasts from patients and controls treated with Tf-Alexa Fluor-488. Nuclei stained with Topro 3 are shown in red. Representative fields are shown. B. Statistical analysis of fluorescence intensity of Tf-Alexa Fluor-488/cell. In all experiments, samples from 5 subjects in each group of patients and controls were examined. Three independent experiments were performed using samples from each group

Supplementary figure 9:

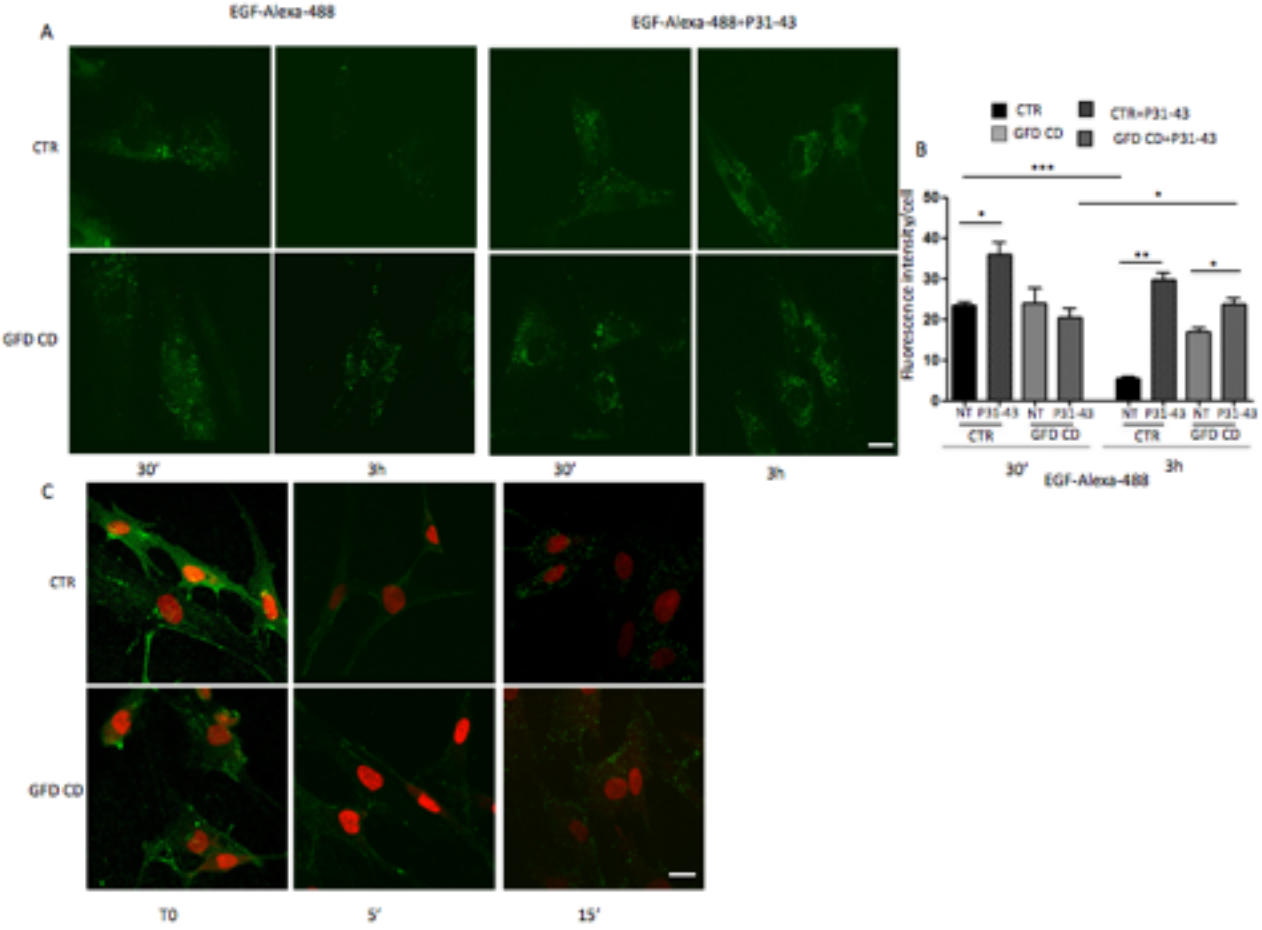

Supplementary figure 9:

Trafficking of EGF-Alexa Fluor-488 in skin fibroblasts from patients with CD and controls cultured in the absence and presence of P31-43. A. EGF-Alexa Fluor-488 decay was delayed in CD fibroblasts and treatment with P31-43 induced a delay in EGF-Alexa Fluor-488 decay in control fibroblasts. Fibroblasts from patients with CD and controls were pulsed with EGF-Alexa Fluor-488 for 1 h at 4°C (not shown) and chased with unlabelled EGF for 30 min or 3 h to monitor the degradation of the EGF/EGFR complex. B. Skin fibroblasts from patients with CD on a GFD and controls were treated with P31-43 for 10’, pulsed with EGF-Alexa Fluor-488 for 1 h at 4°C (not

shown) and chased with unlabelled EGF at 37°C for 30’ or 3 h to monitor EGF-Alexa Fluor-488 decay. Representative fields are shown. Scale bar = 20µm. C. Statistical analysis of the fluorescence intensity of EGF-Alexa Fluor-488. In control fibroblasts, but not cells from patients with CD, EGF-Alexa Fluor-488 levels were decreased after a 3 h chase. After treating cells from both patients and controls with P31-43 for 3 h, EGF-Alexa Fluor-488 levels were increased compared with untreated samples. Three independent experiments were performed on samples from 5 patients and 5 controls. Columns represent the means and bars represent standard deviations. Student’s t-test: * $P<0.05$, ** $P<0.01$, *** $P<0.001$.

D. EGF-Alexa Fluor-488 uptake was similar in CD and control fibroblasts. Fibroblasts from patients with CD and controls were pulsed with EGF-Alexa Fluor-488 for 1 h at 4°C and chased at 37°C for 5 and 15 minutes. After an acid wash (250 mM NaCl, 10 mM HCl), the cells were processed as described in the Methods section. EGF-Alexa Fluor-488 is shown in green, and nuclei stained with Topro 3 are shown in red. Representative fields are shown. Three independent experiments using samples from each of the 5 patients and 5 controls were performed, and similar results were obtained.

Supplementary figure 10:

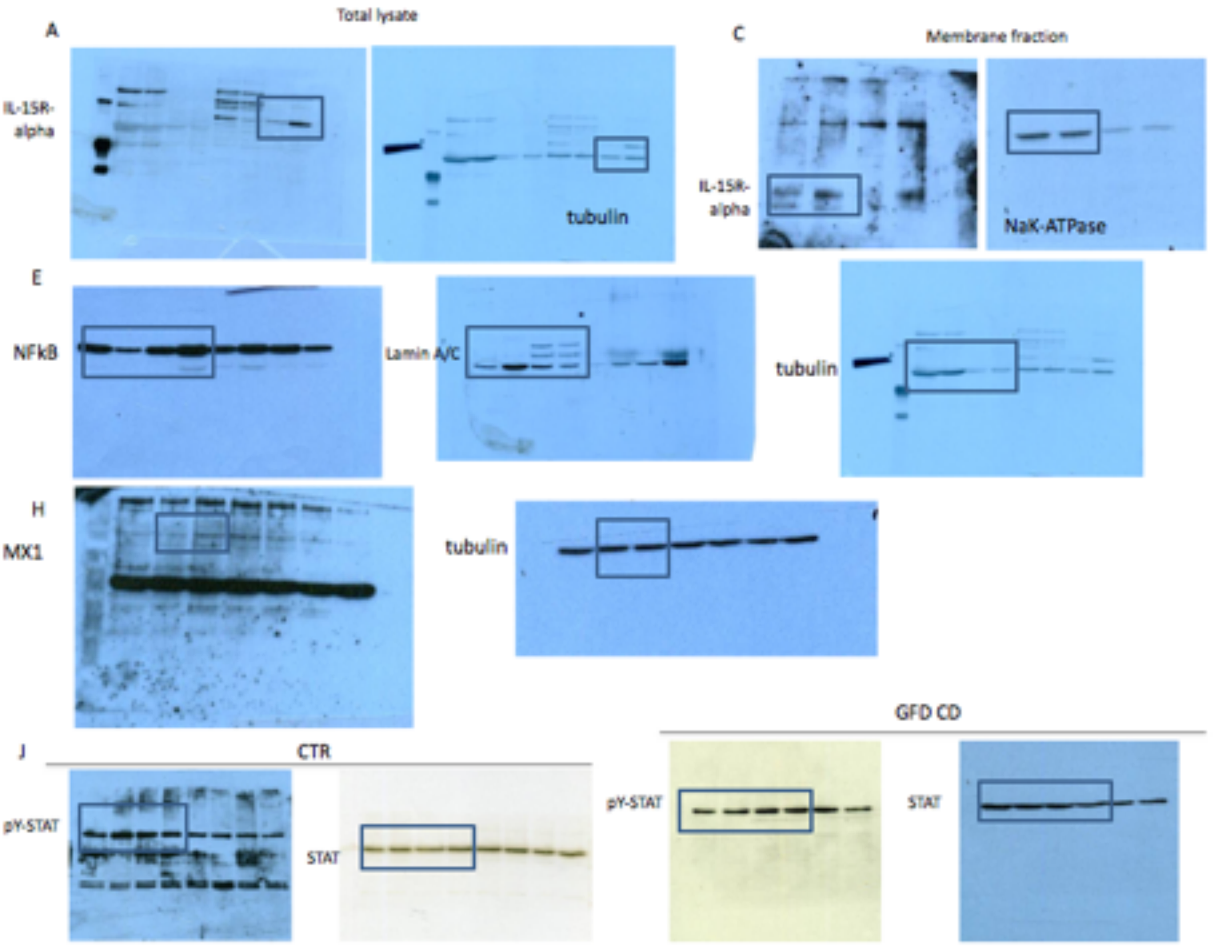

Supplementary figure 10: Un-cropped Blots from figure 6. The letters (A, C,E,H,J) on the un-cropped gels refers to that of the figure 6.

Supplementary figure 11

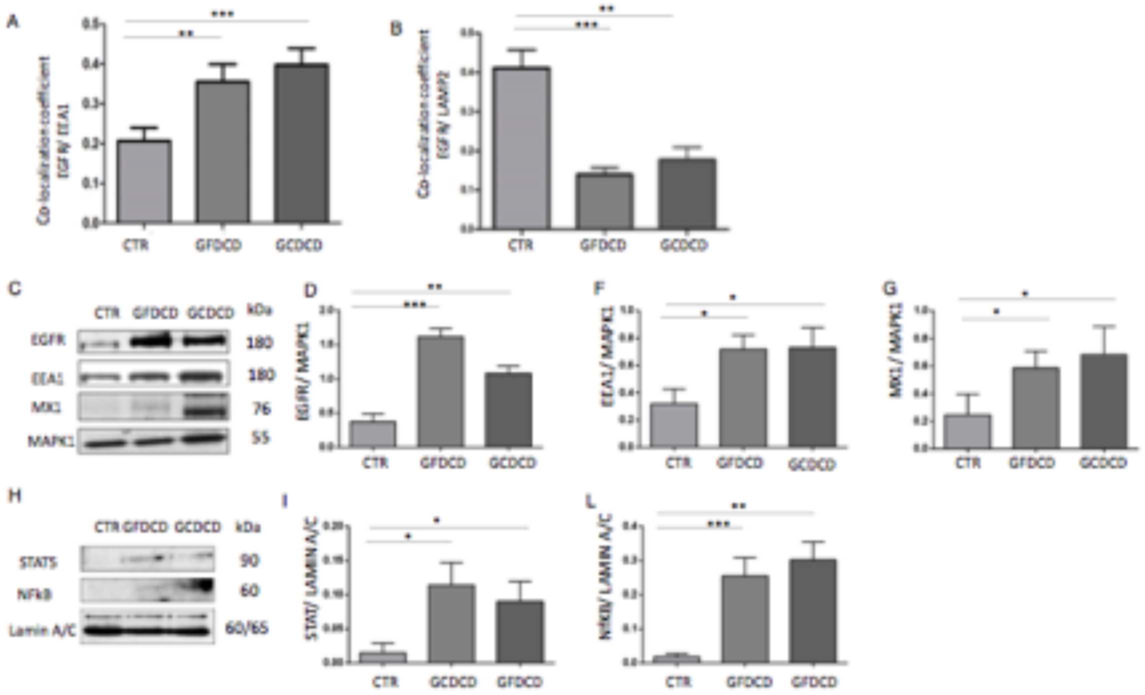

Supplementary figure 11

CD patients (GCD CD and GFD CD) fibroblasts derived from intestinal biopsies presented the same celiac cellular phenotype found in skin fibroblasts.

A. EGFR was localized more in the early vesicular compartment and less in the late vesicular compartment of intestinal fibroblasts from patients with CD respect to CTR. In CD fibroblasts compared to controls, greater amount of EGFR co-localized with EEA1- (A) and less with LAMP2- (B) positive vesicles. Statistical analysis of the co-localization coefficient of EGFR with EEA1 or LAMP2 staining. In all experiments, samples from 3 patients and 3 controls were tested. Columns represent the means, and bars represent standard deviations. Student's t-test,** $P<0.01$, *** $P<0.001$. C-G. Levels of the EGFR, EEA1 and MX1 proteins are increased in intestinal fibroblasts from patients with CD (GCD CD and GFD CD) respect to controls (CTR). C. WB of protein lysates of intestinal fibroblasts, blotted with anti-EGFR, anti-EEA1, anti-MX1, and anti-MAPK1 antibodies used as a loading control. D-G. Densitometry analysis of samples from GCD CD, GFD CD and CTR. In all experiments, samples from 3 subjects in each group of patients and controls were examined. Columns represent the means, and bars represent standard deviations; Student's t-test, * $P<0.05$, ** $P<0.01$, *** $P<0.001$. H-L. Innate immunity and inflammation markers are increased in intestinal fibroblasts from patients with CD (GCD CD and GFD CD) respect to controls (CTR). Levels of the innate immunity marker STAT5 and the inflammation marker NFκB are increased in CD fibroblasts from intestinal biopsies. H. WB analysis of proteins from nuclear fraction of CTR and CD fibroblasts blotted with anti-STAT5 and NFκB antibodies, as indicated. LaminA/C was used as nuclear fraction loading control. Representative blots. I, L. Densitometry analysis of WBs of samples from 3 patients and 3 controls. Columns represent the means and bars represent standard deviations. Student's t-test compared to the not treated (NT) sample, * $P<0.05$, ** $P<0.01$, *** $P<0.001$.

Supplementary figure 12

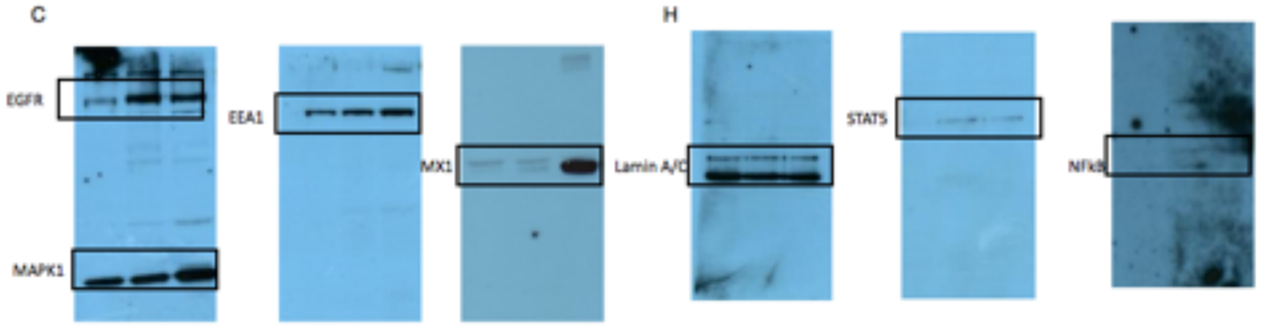

Supplementary figure 12: Un-cropped Blots from Supplementary figure 11. The letters (C and H) on the un-cropped gels refers to that of the supplementary figure11.

Supplementary figure 13

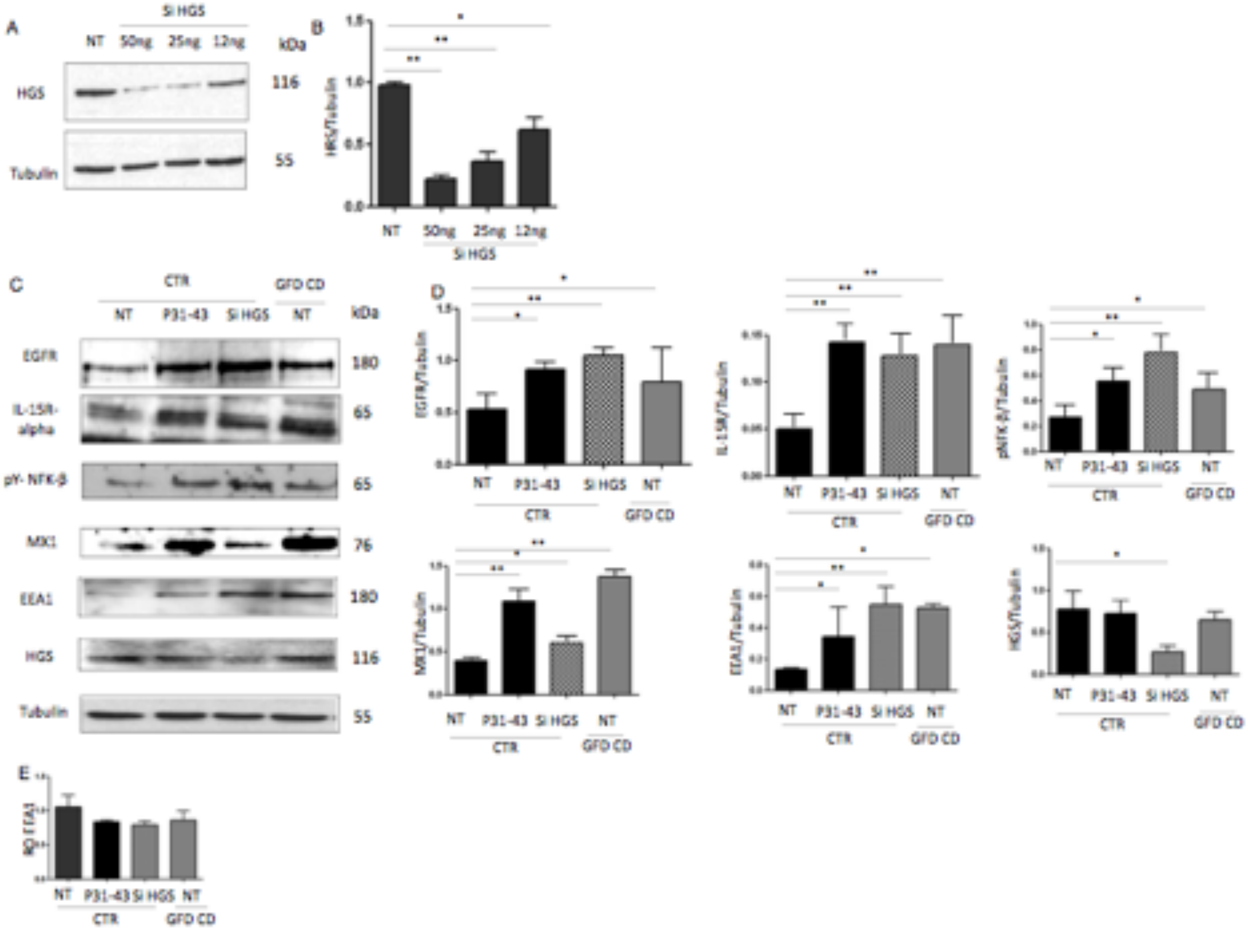

Supplementary figure 13

A delay in early endocytic vesicles maturation obtained by HGS silencing induced in control skin fibroblasts the celiac cellular phenotype.

Modulation of HGS protein levels by silencing with different siRNA concentrations. A. and B Western blot of protein lysates from CTR fibroblasts before and after HGS silencing at different concentrations as indicated and blotted with anti-HGS and anti-tubulin antibodies. Reprentative blot. B. Densitometry analysis. In all experiments, samples from 4 controls and 4 patients were examined. C and D. HGS silencing and P31-43 treatment increased levels of the EGFR, IL15R-α, MX1, EEA1 proteins and NFκB phosphorylation in control fibroblasts. C. Western blot analysis of protein lysates from CTR fibroblasts before and after HGS silencing and P31-43 treatment, as well as from CD fibroblasts, blotted with anti-EGFR, anti IL15R-α, anti-pY-NFκB, anti-MX1, anti-EEA1, anti-HGS and anti-tubulin antibodies, as indicated. Representative blots are shown. D. Densitometry analysis. HGS (50ng siHGS) silencing decreased levels of the HGS protein by 67±14%. HGS silencing as well as P31-43 treatment (30 min) increased levels of the EGFR, IL15R-α, MXA and EEA1 proteins and NFκB phosphorylation in control fibroblasts. In all experiments, samples from 4 controls and 4 patients were examined. Columns represent the means and bars represent standard deviations. Student's t-test, * $P<0.05$, ** $P<0.01$. E. The quantitative PCR analysis showed that mRNA levels of the EEA1 mRNA were not increased in CD fibroblasts or control cells after P31-43 treatment or siHGS transfection. Columns represent the means, and bars represent the standard deviations of a representative experiment performed in triplicate. Similar results were obtained from samples from 4 patients and 4 controls.

Supplementary figure 14

A

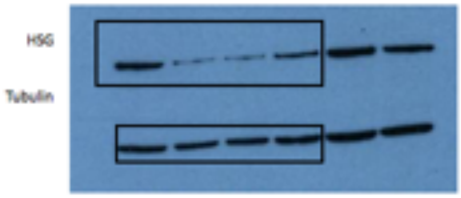

C

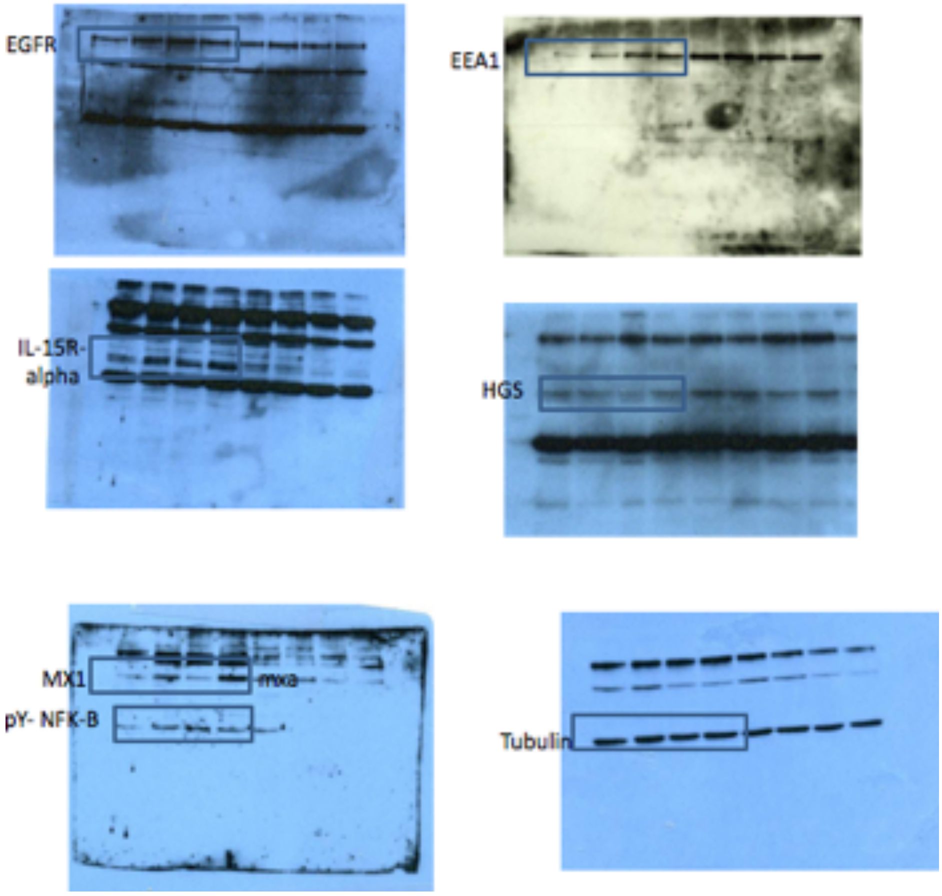

Supplementary figure 14: Un-cropped Blots from Supplementary figure 13. The letter (A and C) on the un-cropped gels refers to that of the supplementary figure13.

Supplementary figure 15

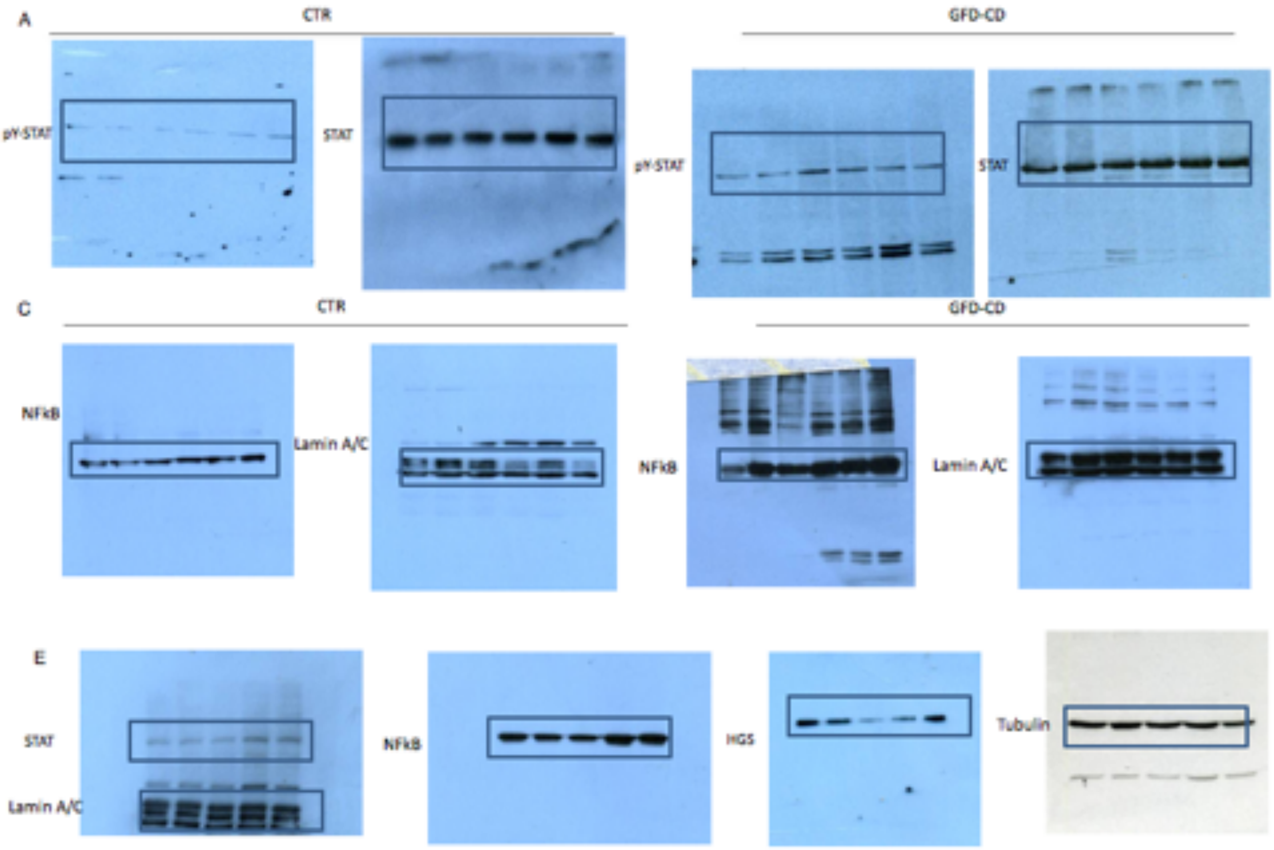

Supplementary figure 15: Un-cropped Blots from figure 9. The letters (A, C,E,) on the un-cropped gels refers to that of the figure 9.

Supplementary figure 16

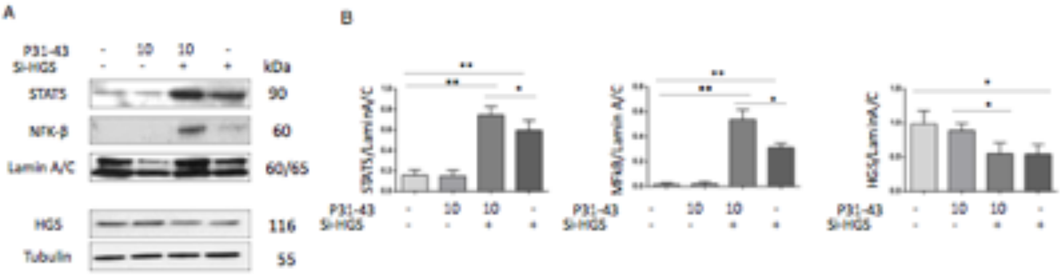

Supplementary figure 16

Effects of very low doses of siHGS and P31-43 on STAT 5 and NFκB activation in skin fibroblasts from GFD patients. Very low amount of siHGS and P31-43 activated STAT 5 and NFκB in CD skin fibroblasts. STAT 5 and NFκB nuclear fraction in response to treatment with inactive (10 µg/ml) doses of P31-43 in GFD-CD skin fibroblasts before and after silencing HGS (12ng siHGS). A. WB analysis of proteins from nuclear fraction blotted with antibodies anti-STAT 5 and anti-NFκB and anti-lamin A/C (nuclear loading control) and of proteins from cytosol fraction blotted with antibodies anti-HGS, anti-tubulin (citosol loading control), as indicated. Representative blots are shown. B. Densitometry analysis of WBs of samples from 3 GFD-CD patients. Columns represent the means, and bars represent standard deviations. Student's t-test compared to the not treated (NT) sample, * $P<0.05$, ** $P<0.01$.

Supplementary figure 17

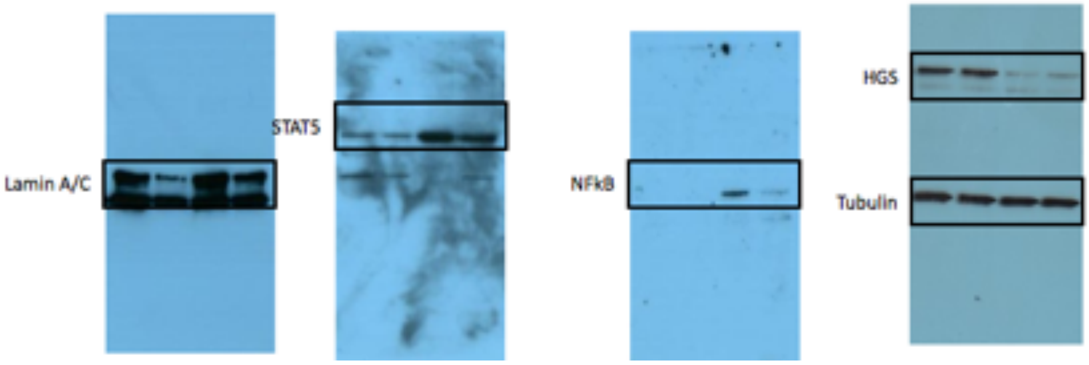

Supplementary figure 17: Un-cropped Blots from Supplementary figure 13.

Patients	Mean Age (Years)	Sex	Biopsy (Marsh classification*)	Serum Anti-TG2 (U/ml)	Anti-Endomysial Antibody (EMA)
Controls (N=12)	6	M=4<br>F=8	T0	0-1.5	Negative
GCD-CD(N=12)	5	M=3<br>F=9	10=T3c<br>2=T3cb	>50	Positive
GFD-CD (N=10)	12	M=4<br>F=6	T0	0-1.5	Negative

* * T0: Normal; T1: infiltrative lesion; T3: Flat destructive lesion (a:mild, b:moderate, c:total)

Supplementary Table 1. Patient information

Supplementary Material and Methods

Co-localization analysis

Samples were examined with a Zeiss LSM 510 laser scanning confocal microscope. We used Argon/2 (458, 477, 488, 514 nanometers) and HeNe1 (543 nanometers) excitation lasers, which were switched on separately to reduce cross-talk of the two fluorochromes. The green and the red emissions were separated by a dichroic splitter (FT 560) and filtered (515-to 540-nm band-pass filter for green and 610-nm long pass filter for red emission). A threshold was applied to the images to exclude about 99% of the signal found in control images. The weighted co-localisation coefficient represents the sum of intensity of co-localising pixels in channels 1 and 2 as compared to the overall sum of pixel intensities above threshold. This value could be 0 (no colocalisation) or 1 (all pixels co-localise). Bright pixels contribute more than faint pixels. The co-localisation coefficient represents the weighted co localisation coefficients of Ch1 (red) with respect to Ch2 (green) for each experiment (1,2). In the GCD biopsies, when the villi were too flat, the surface epithelium, between the crypts, was evaluated.

Primary fibroblast culture.

Skin explants or duodenal biopsies were immediately placed in Dulbecco's Modified Eagle's Medium (DMEM) (GIBCO, San Giuliano Milanese, Italy) supplemented with 20% foetal bovine serum (FBS) (GIBCO, San Giuliano Milanese, Italy), 100 units/ml penicillin-streptomycin (GIBCO, San Giuliano Milanese, Italy), and 1 mM glutamine (GIBCO, San Giuliano Milanese, Italy) and incubated for 24 hours. Subsequently, each skin or duodenal explant was divided into about approximately 50 small fragments, plated on T 25 flasks and incubated in the presence of 95% oxygen and 5% CO_2 at 37°C to allow adhesion and the subsequent release of fibroblasts.

Seven-ten days later, the fibroblasts began to emerge from the fragments. When the fibroblasts reached confluence, the cells were harvested with trypsin and immediately frozen. In all experiments, the fibroblasts were used between the 2nd and 4th passage.

Immunofluorescence staining of fibroblasts

Fibroblasts were seeded on glass coverslips and then starved in starvation medium (DMEM, 0.5% FBS, 100 units/ml penicillin-streptomycin and 1 mM glutamine) after 2 days to construct EGFR degradation curves. After approximately 18 h, the control coverslips were collected and fixed. Afterwards, EGF (100 ng/ml) was added, to the coverslips and they were incubated for different periods (30', 1 h, 3 h, 6 h and overnight (ON)) and then fixed. In all cases, cells were fixed with 3% paraformaldehyde (PFA, Sigma-Aldrich, Milan, Italy) for 5 min and then permeabilized with 0,1% Triton for 5 min. Coverslips were than washed 3 times with PBS and double staining was performed with mouse anti-EGFR antibodies (F-9 sc-37707, Santa Cruz Biotechnology, CA, USA) and a goat anti-EEA1 antibody (sc-6414, Santa Cruz Biotechnology, CA, USA) for 1 h at room temperature in a dark chamber. Coverslips were then washed (3 times, 5 min each) with PBS at RT, and fluorescein-conjugated anti-rabbit (for EGFR) (1,50, SC-03, Santa Cruz Biotechnology, CA, USA) and anti-goat secondary antibodies (for EEA1) (1,50, Santa Cruz Biotechnology, CA, USA) were applied for 45 min at room temperature in a dark box to avoid bleaching. After washes with PBS, cells were counterstained with Hoechst (Sigma-Aldrich, Milan, Italy), mounted with Mowiol (Sigma-Aldrich, Milan, Italy) and observed under a Zeiss LSM 510 confocal microscope (Germany). The co-localization analysis was performed as described above.

For transferrin recycling experiments, fibroblasts were serum-starved for 1 h at 37°C, washed twice with cold phosphate-buffered saline (PBS) containing 1% BSA (bovine serum albumin, Sigma-Aldrich, Milan, Italy), and then exposed to 100 µg/ml Alexa-Fluor-488-Tf (1,100, Invitrogen, San Giuliano Milanese, Italy) for 1 h at 37°C. After extensive washes with complete HEPES-buffered

DMEM, Tf recycling was monitored by incubating the cells with complete medium for 60 min at 37°C. Samples were then fixed and observed under a confocal microscope (LSM 510 Zeiss). For transferrin uptake assays, skin fibroblasts were continuously incubated with 50 µg/ml Tf-Alexa-Fluor-488 in complete HEPES-buffered DMEM for 2 h at 37°C, washed with an acidic solution (150 mM NaCl and 10 mM acetic acid, pH 3.5), and then fixed. Images were obtained with a 63X objective unless differently stated.

Western Blot

The lysates from biopsies and fibroblasts were washed twice in PBS and resuspended in lysis buffer (Tris-HCl, pH 7.4, 50 mM, EDTA 1 mM, EGTA 1 mM, 5 mM $MgCl_2$, 150 mM NaCl, 1% Triton,1 mM PMSF, 1 mM VO_4, Aprotinin, and LAP, all purchased from Sigma, Milan, Italy, except LAP, which was obtained from Roche, Milan, Italy). The cell lysates were analyzed with SDS-PAGE using running buffer (25 mM Trizma, 192 mM Glycine, and 0.1% SDS) and transferred onto nitrocellulose membranes (Whatman Gmbh, Dassel, Germany) using transfer buffer (25 mM Trizma, 192 mM Glycine, 0.1% SDS, and 20% methanol, all purchased from Sigma-Aldrich, Milan, Italy).

For the separation of membranes and nuclei from cytosol, near-confluent fibroblasts were incubated in a 100-mm dish containing 1 ml of homogenization buffer (10 mM Tris-HCl [pH 7.4], 1 mM EDTA, 1 mM PMSF, 1 mM VO_4, Aprotinin, and LAP; all purchased from Sigma, Milan, Italy, except for LAP, which was purchased from Roche, Milan, Italy). The nuclear fraction was separated through centrifugation at 1,000g (postnuclear supernatant and nuclear fraction), and the pellet was incubated in 100 µl of homogenization buffer containing 0.4 M NaCl for 1 h on ice to disrupt the nuclei. After centrifugation at 105,0006g for 1 h, the supernatant (the crude nuclear extract) was recovered. The post-nuclear supernatant was centrifuged at 105,0006g for 1 h, and the cytosolic fraction (supernatant) was separated. The pellet (membranes) was incubated in 50 µl of homogenization buffer containing 0.5% Nonidet P-40 for 1 h on ice, centrifugation at 105,0006g

for 1 h. The cytosolic, the crude nuclear extract and membranes were analyzed using immunoblotting.

The membranes were subsequently blocked using 5% non-fat dry milk solution and probed with antibodies anti-EEA1 (Santa Cruz Biotechnology CA, USA), anti-TfR (Santa Cruz Biotechnology, CA, USA) anti-LAMP2 , anti-EGFR (Cell Signalling, Euroclone Milan, Italy), anti-IL15R alpha (Santa Cruz Biotechnology CA, USA), anti-STAT 5 (Cell Signalling, Euroclone Milan, Italy) anti-MX1 (Santa Cruz Biotechnology CA, USA), anti-HGS mouse (Alexis, Vinci-Biochem, Florence, Italy) anti-NaKATPase (Cell Signalling, Euroclone Milan, Italy) antibodies. The membranes were washed, blocked with 5% non-fat dry milk and probed with anti-pTyr-STAT 5 (Cell Signalling, Euroclone Milan, Italy), anti-pTyr-NFKB (Cell Signalling, Euroclone Milan, Italy). Anti-tubulin mouse monoclonal antibody (SIGMA-Aldrich, Milan, Italy) and anti-MAPK1 were used as loading controls for fibroblasts and biopsies respectively.

The bands were visualized using ECL (GE Healthcare, Amersham, Buckinghamshire, UK) and exposure times of 2– 10 min. The band intensity was evaluated by integrating all of the pixels of the band without the background to calculate the average of the pixels surrounding the band (1).

Electro Microscopy

Fibroblasts were grown in 10-cm petri dishes and fixed with 1% glutaraldehyde in 0.15 M Hepes for 2 h at room temperature. Samples were then stained with OsO4/potassium ferrocyanide, dehydrated and embedded in epon, as described previously (3). Ultrathin sections (60-70 nm) were obtained, examined using a Tecnai-12 (FEI, Eindhoven) electron microscope, and images were acquired using a Veletta CCD digital camera. Random sampling of cellular profiles for the endocytic organelles was performed and the structures were classified based on the following morphological criteria, 1) endocytic structures with less than 8 intraluminal vesicles were classified as early endosomes (EE) and 2) the remaining endocytic structures (with more than 8 intraluminal vesicles or other membranes) were classified as lysosome-like structures (LLS) (3).

1. Barone, M.V. et al. Growth factor-like activity of gliadin, an alimentary protein, implications for celiac disease. *Gut* **56**, 480-8 (2007).

2. Barone, M.V. et al. Gliadin peptide P31-43 localises to endocytic vesicles and interferes with their maturation. *PloS One* **5**, e12246 (2010).

3. Rizzo, R. et al. The dynamics of engineered resident proteins in the mammalian Golgi complex relies on cisternal maturation. *J. Cell Biol.* **201**, 1027-36 (2013).
