# Supplementary material for: Constitutive alterations in vesicular trafficking increase the sensitivity of cells from celiac disease patients to gliadin
Source: Commun Biol. 2019 May 20;2:190. doi: 10.1038/s42003-019-0443-1 (PMC6527696; doi:10.1038/s42003-019-0443-1)
Supplement: Supplementary file 3 — Reporting Summary [file 42003_2019_443_MOESM3_ESM.pdf]

nature research

Corresponding author(s): BDPR COMMSBIO-18-0683B

Last updated by author(s): 2019 04 02

Reporting Summary

Nature Research wishes to improve the reproducibility of the work that we publish. This form provides structure for consistency and transparency in reporting. For further information on Nature Research policies, see Authors & Referees and the Editorial Policy Checklist.

Statistics

For all statistical analyses, confirm that the following items are present in the figure legend, table legend, main text, or Methods section.

n/a	Confirmed	
☐	☒	The exact sample size (*n*) for each experimental group/condition, given as a discrete number and unit of measurement
☐	☒	A statement on whether measurements were taken from distinct samples or whether the same sample was measured repeatedly
☐	☒	The statistical test(s) used AND whether they are one- or two-sided<br>*Only common tests should be described solely by name; describe more complex techniques in the Methods section.*
☐	☒	A description of all covariates tested
☐	☒	A description of any assumptions or corrections, such as tests of normality and adjustment for multiple comparisons
☐	☒	A full description of the statistical parameters including central tendency (e.g. means) or other basic estimates (e.g. regression coefficient) AND variation (e.g. standard deviation) or associated estimates of uncertainty (e.g. confidence intervals)
☐	☒	For null hypothesis testing, the test statistic (e.g. *F*, *t*, *r*) with confidence intervals, effect sizes, degrees of freedom and *P* value noted<br>*Give P values as exact values whenever suitable.*
☒	☐	For Bayesian analysis, information on the choice of priors and Markov chain Monte Carlo settings
☒	☐	For hierarchical and complex designs, identification of the appropriate level for tests and full reporting of outcomes
☒	☐	Estimates of effect sizes (e.g. Cohen's *d*, Pearson's *r*), indicating how they were calculated

Our web collection on statistics for biologists contains articles on many of the points above.

Software and code

Policy information about availability of computer code

Data collection	NA
Data analysis	NA

For manuscripts utilizing custom algorithms or software that are central to the research but not yet described in published literature, software must be made available to editors/reviewers. We strongly encourage code deposition in a community repository (e.g. GitHub). See the Nature Research guidelines for submitting code & software for further information.

Data

Policy information about availability of data

All manuscripts must include a data availability statement. This statement should provide the following information, where applicable:

- Accession codes, unique identifiers, or web links for publicly available datasets
- A list of figures that have associated raw data
- A description of any restrictions on data availability

All data generated or analysed during this study are included in this published article (and its supplemental information files)

Field-specific reporting

Please select the one below that is the best fit for your research. If you are not sure, read the appropriate sections before making your selection.

☒ Life sciences ☐ Behavioural & social sciences ☐ Ecological, evolutionary & environmental sciences

Life sciences study design

All studies must disclose on these points even when the disclosure is negative.

Sample size	The samples were randomly chosen avoiding subjects affected by other concomitant diseasese. Considering the complexity of the analysis and the differences between affected and not affected subjects we reguarded the number to be sufficient.
Data exclusions	No data were excluded
Replication	All data were reproducible
Randomization	Samples have been randomly selected and accurately represent their respective populations.
Blinding	All fluorescence data were observed by 2 researchers, blindly.

Reporting for specific materials, systems and methods

We require information from authors about some types of materials, experimental systems and methods used in many studies. Here, indicate whether each material, system or method listed is relevant to your study. If you are not sure if a list item applies to your research, read the appropriate section before selecting a response.

Materials & experimental systems

n/a	Involved in the study	
☐	☒	Antibodies
☐	☒	Eukaryotic cell lines
☒	☐	Palaeontology
☒	☐	Animals and other organisms
☒	☐	Human research participants
☐	☒	Clinical data

Methods

n/a	Involved in the study	
☒	☐	ChIP-seq
☒	☐	Flow cytometry
☒	☐	MRI-based neuroimaging

Antibodies

Antibodies used	For all the antibodies name, clone and manifacturer is described
Validation	All the antibodies have a reference

Eukaryotic cell lines

Policy information about cell lines

Cell line source(s)	Skin explants or duodenal biopsies were immediately placed in Dulbecco's Modified Eagle's Medium (DMEM) (GIBCO, San Giuliano Milanese, Italy) supplemented with 20% foetal bovine serum (FBS) (GIBCO, San Giuliano Milanese, Italy), 100 units/ml penicillin-streptomycin (GIBCO, San Giuliano Milanese, Italy), and 1 mM glutamine (GIBCO, San Giuliano Milanese, Italy) and incubated for 24 hours. Subsequently, each skin or duodenal explant was divided into about approximately 50 small fragments, plated on T 25 flasks and incubated in the presence of 95% oxygen and 5% CO2 at 37°C to allow adhesion and the subsequent release of fibroblasts. Seven-ten days later, the fibroblasts began to emerge from the fragments. When the fibroblasts reached confluence, the cells were harvested with trypsin and immediately frozen. In all experiments, the fibroblasts were used between the 2nd and 4th passage.
Authentication	Authentication was done by vimentine staining and fluorescence microscope observation. All the fibroblasts were positive for this marker
Mycoplasma contamination	All the cell used in the study were tested for micoplasma contamination by Hoechst staining and fluorescence microscope observation
Commonly misidentified lines (See ICLAC register)	NA

Clinical data

Policy information about clinical studies

All manuscripts should comply with the ICMJE guidelines for publication of clinical research and a completed CONSORT checklist must be included with all submissions.

Clinical trial registration	NA. Our study is not a clinical trial
Study protocol	NA
Data collection	NA
Outcomes	NA
